# Photobiomodulation with 808 nm Laser Light Repairs Mitochondrial Integrity and Normalizes ROS, ATP, and Membrane Depolarization in Aβ-Exposed Primary Neurons in Alzheimer’s Disease Model

**DOI:** 10.3390/ijms27177834

**Published:** 2026-09-01

**Authors:** Iuliia Golovynska, Binjun Li, Qinglin Chen, Sergii Golovynskyi, Hao Xu, Yurii V. Stepanov, Liudmyla I. Stepanova, Fangrui Lin, Junle Qu, Tymish Y. Ohulchanskyy

**Affiliations:** 1 Shenzhen Key Laboratory of Photonics and Biophotonics, College of Physics and Optoelectronic Engineering, Shenzhen University, Shenzhen 518060, China; iuliia@szu.edu.cn (I.G.); tyo2@buffalo.edu (T.Y.O.); 2 R.E. Kavetsky Institute of Experimental Pathology, Oncology and Radiobiology, National Academy of Science of Ukraine, 03022 Kyiv, Ukraine; 3 Institute of Biology and Medicine, Taras Shevchenko National University of Kyiv, 01601 Kyiv, Ukraine

**Keywords:** photobiomodulation, amyloid-β, Alzheimer’s disease, mitochondrial network morphology, neurons

## Abstract

Alzheimer’s disease (AD) is increasingly recognized as a disorder involving profound mitochondrial dysfunction. Although photobiomodulation (PBM) has shown neuroprotective efficacy in experimental AD models, whether restoration of mitochondrial architecture is mechanistically required for these effects remains unknown. Here, we investigated the role of mitochondrial network remodeling in PBM-mediated neuroprotection in primary mouse hippocampal neurons exposed to amyloid-β (Aβ). Neurons were treated for 24 h with oligomeric Aβ_1–42_ and irradiated with 808 nm PBM (100 mW/cm^2^, 30 J/cm^2^). Mitochondrial morphology was quantified using three-dimensional confocal microscopy and computational network analysis. Aβ exposure induced severe mitochondrial fragmentation and swelling, reflected by increased mitochondrial count and sphericity together with reduced mitochondrial volume, surface area, branch length, and network connectivity. These structural alterations were accompanied by elevated reactive oxygen species production, adenosine triphosphate depletion, membrane depolarization, and reduced neuronal viability. PBM significantly reversed these abnormalities, restoring mitochondrial network integrity and partially normalizing cellular bioenergetics and redox homeostasis. To determine whether mitochondrial dynamics contributes to PBM-induced effects, neurons in another experiment were pre-treated with the dynamin-related protein-1 (DRP-1) mitochondrial division inhibitor-1 (Mdivi-1). The physiological and morphological profiles of the Aβ + PBM + Mdivi-1 and Aβ + Mdivi-1 groups were found to be largely indistinguishable in this case, revealing that PBM failed to restore mitochondrial connectivity, cellular bioenergetics, or viability of neurons in the partially blocked fission–fusion machinery. These findings demonstrate that mitochondrial dynamics is essential for PBM-mediated neuroprotection and identify restoration of mitochondrial network integrity as a crucial mechanism linking PBM to improved neuronal bioenergetics, redox balance, and survival in AD.

## 1. Introduction

Alzheimer’s disease (AD) causes a gradual decline in cognitive abilities. Clinical features observed in the AD brain include intracellular neurofibrillary tangles, composed of hyperphosphorylated tau protein (p-tau), and extracellular amyloid-β (Aβ) plaques (a core pathological stimulus of AD), as well as overall brain tissue shrinkage and degradation [1,2]. A comprehensive understanding of the AD origins does not currently exist; research interest is focused on different mechanisms, including those involving mitochondria [3,4,5]. Mitochondrial dysfunction is currently emerging as a potential marker for early detection of AD progression. It is linked to decreased production of adenosine triphosphate (ATP), excessive formation of reactive oxygen species (ROS), and disruptions in mitochondrial dynamics, such as fission and fusion [4,6,7,8].

Accumulating clinical and preclinical evidence strongly confirms that mitochondrial dynamics are profoundly disturbed in the pathological progression of AD. Specifically, mitochondrial structural damage is mainly driven by Aβ exposure, which triggers extensive mitochondrial fragmentation, swelling, reduced branching complexity, and impaired network connectivity in neurons [9,10]. These prominent mitochondrial architectural abnormalities are further underpinned by the dysregulation of core regulatory proteins that govern mitochondrial fusion and fission dynamics, including the fusion mediators mitofusin-1 (MFN-1), mitofusin-2 (MFN-2), and optic atrophy protein-1 (OPA-1), as well as the key fission regulator dynamin-related protein-1 (DRP-1) [11]. Consistent with these findings, numerous studies have highlighted the significant role of disturbances in mitochondrial network morphology in AD development [10,11,12]. An assessment of various morphological characteristics of the mitochondrial network, such as length, number of branches, and degree of connectivity, has shown that mitochondria are fragmented and swollen as a result of Aβ influence [9]. The fragmented mitochondrial networks show a slower transfer of substances within their structure in comparison with the branched and connected ones [13]. This phenomenon is also associated with impaired mitophagy, which hampers the removal of damaged mitochondria [14]. On the other hand, extracellular Aβ plaques precede the spread of p-tau neurofibrillary tangles across limbic and cortical circuits in human AD brains [3], and p-tau independently disrupts mitochondrial transport, impairs mitochondrial fusion proteins MFN-1/2 and OPA-1, amplifies fission via DRP-1 upregulation, and exacerbates oxidative stress and ATP depletion through microtubule destabilization [15].

Given the long lifespan and the lack of proliferation of neurons, they are unable to distribute defective mitochondria among newly formed cells and therefore, in contrast to dividing cells, rely primarily on fusion/fission mechanisms to maintain or restore the functionality of damaged mitochondria [16]. The persistent disruption of mitochondrial structural integrity and dynamic balance ultimately exacerbates mitochondrial functional failure, and these cumulative damaging disturbances synergistically drive AD pathological progression, eventually leading to irreversible neuronal death [4]. However, despite increasing recognition of the importance of mitochondrial architecture in neuronal health, relatively little is known about therapeutic approaches capable of restoring mitochondrial network integrity under AD-related conditions.

Red and near-infrared (NIR) photobiomodulation (PBM) therapy is being actively used as a therapeutic approach for neurodegenerative diseases, especially AD [17,18,19,20,21,22,23,24,25,26,27,28]. It has been shown that PBM of AD mice can mitigate the creation of Aβ plaques [18,19,20,21,22,23,24,25,26,27,28], Aβ-induced cell membrane disruptions (lipid disordering and rafts) [26], neuronal damage [24,25,26,27,28,29], and microgliosis [20,21,22,27,29] within transgenic animal models. Moreover, it aids in slowing down the progression of neurodegenerative conditions [22,28,30,31,32]. Multiple clinical trials evaluate PBM for AD. Conventional PBM approaches include transcranial helmet-based [19,20,33] and intranasal [34,35,36] irradiations, both of which bypass skull attenuation to target vulnerable limbic and cortical brain regions, with randomized controlled trials reporting improved memory and reduced neuropsychiatric symptoms. An emerging alternative targets the gut–brain axis via abdominal PBM. Pilot clinical trials using daily abdominal red/NIR irradiation demonstrate meaningful cognitive gains, driven by modulated gut microbiota, enhanced intestinal barrier function, vagus nerve anti-inflammatory signaling, and indirect central mitochondrial protection [37,38]. The prospect is dual head and abdominal PBM therapy, with multi-center pivotal effects. Direct cranial PBM can exert rapid neuronal rescue, while abdominal PBM can rely on systemic gut-derived signaling, representing complementary translational strategies for AD treatment [39].

The interaction of light with biological cells leads to different photochemical reactions. Red/NIR light within the wavelength range of ~600–980 nm is absorbed by the cytochrome c oxidase (CCO, mitochondrial complex IV) photoacceptor, located in mitochondria [40,41,42]. A photoexcitation of CCO leads to the elevation of mitochondrial membrane potential (MtMP) [43,44,45,46]. Nitric oxide (NO) competitively binds to the O_2_ active site of CCO and suppresses electron transport chain (ETC) flux. One of the major mechanisms of red/NIR-PBM is photon-induced photodissociation of inhibitory CCO–NO complex, relieving NO-mediated respiratory chain suppression and restoring CCO catalytic activity [47]. Various intracellular pathways are consequently also affected via light-modulated ROS production [41,42], ATP synthesis [43,48,49,50], and caspase family members activation [51,52]. On the other hand, red/NIR light can depolarize cell membrane potential (CMP), leading to the activation of calcium ion (Ca^2+^) channels and the endoplasmic reticulum (ER) [53,54,55,56]. These redox-based and Ca^2+^ signaling contribute to upsurge mitochondrial oxidative stress, alongside the regulation of antioxidant genes, facilitating cellular proliferation and enhancing survival rates [25,29,57,58,59]. What is important to AD therapy, these signalings result in the alleviation of Aβ via the downregulation of genes supporting AD development, such as those responsible for the expression of amyloid precursor protein, β-/γ-secretase, and others [23,26,60]. On the other hand, genes involved in oxidative stress, inflammation, apoptosis, and cholesterol homeostasis are also modulated [26,61].

During the last several years, the mechanistic aspects underlying red/NIR light-mediated management of AD have been only partially elucidated, with prevailing evidence pointing toward light-triggered suppression of amyloidogenic cellular pathways, oxidative stress, neuroinflammation, and apoptosis [23,26,27,28,29,62]. Light exposure has been shown to consistently modulate mitochondrial activity in both brain neurons [55,63] and peripheral cell types [41,42,43,44,45,46,64,65,66,67,68], increasing MtMP and ROS generation. NIR light has also been reported to instigate changes in mitochondrial dynamics (fission and fusion) during wound healing [69]. In the context of neurodegeneration, growing evidence identifies Aβ-induced mitochondrial abnormalities and differentiation as a critical driver of AD pathogenesis [4,9,70]. On the other hand, it has been recently revealed that PBM modulates mitochondrial energy metabolism and ameliorates neurological damage in AD mice [28,66]. Notably, despite these advances, PBM-induced remodeling of mitochondrial network, including structural changes in either healthy or Aβ-damaged neurons, remains entirely unexplored and unreported. This represents a critical knowledge gap in the current understanding of PBM-mediated neuroprotection against AD.

In this work, the restoration of mitochondrial integrity in primary neurons in vitro after NIR-PBM using an 808 nm laser (100 mW/cm^2^, 5 min, 30 J/cm^2^) is reported. It has been comprehensively studied using three-dimensional (3D) confocal laser scanning fluorescence microscopy (CLSM) followed by the analysis of the obtained CLSM images using imaging analysis software. Mitochondrial morphology was evaluated following the exposure of neurons to Aβ to recapitulate AD-like stress after PBM or without it. Additionally, to determine whether mitochondrial dynamics contributes to PBM-induced effects, neurons in another experiment were pre-treated with a DRP-1 inhibitor. Mitochondrial parameters, such as count, volume, surface area, branches, length, junctions, endpoints, sphericity, and diameter, were quantified. Beyond structural assessments, we quantified critical cellular and metabolic parameters to dissect functional impacts of PBM. These include measured cytosolic ROS (CtROS) and mitochondrial ROS (MtROS) production rates, ATP concentration, and CMP in neurons both under baseline conditions and Aβ exposure and following light irradiation. By integrating structural and functional analyses, this study provides a quantitative framework for understanding how PBM influences mitochondrial organization in neurons and identifies mitochondrial network remodeling as a potential component of PBM-mediated neuroprotection.

## 2. Results

Aβ and p-tau depositions are known to disrupt neuronal functions and cause neuronal damage, ultimately leading to cognitive impairments. Neurons affected by Aβ reveal mitochondrial dysfunction, reduced MtMP, increased ROS generation, oxygen-glucose deprivation, and altered mitochondrial network morphology [3,4,5]. Such Aβ-induced mitochondrial abnormalities and differentiation are pivotal drivers of AD pathogenesis and treatment [9,71,72]. In the current research, hippocampal neurons were exposed to 10 μM soluble oligomeric Aβ_1–42_ for 24 h to establish an in vitro AD model. We utilized an acute Aβ treatment model to rapidly induce robust and quantifiable mitochondrial structural and functional defects for 3D microscopy analysis of mitochondrial network. This acute toxic paradigm differs from clinical AD, where soluble Aβ oligomers accumulate slowly over 10–30 years of prodromal disease. The 10 μM Aβ concentration used herein is substantially higher than picomolar Aβ concentrations measured in cerebrospinal fluid and interstitial fluid of AD patients [73].

We applied NIR-PBM at 100 mW/cm^2^ for 5 min (a resultant dose of 30 J/cm^2^) to treat Aβ-exposed primary neurons in vitro to assess light-induced neuroprotective mechanisms, focusing on changes in mitochondrial network structure. Mitochondrial morphology was evaluated following either 24 h Aβ treatment of neurons, 24 h after 808 nm PBM for 5 min, or after combining both stimuli to the cells. Additionally, to investigate whether mitochondrial network remodeling contributes to the neuroprotective effects of PBM, mitochondrial fission was pharmacologically inhibited using mitochondrial division inhibitor-1 (Mdivi-1, a selective inhibitor of DRP-1). Hence, neurons were allocated to eight experimental groups: (i) untreated control, (ii) PBM alone, (iii) Mdivi-1, (iv) PBM + Mdivi-1, (v) Aβ alone, (vi) Aβ + PBM, (vii) Aβ + Mdivi-1, and (viii) Aβ + PBM + Mdivi-1. For this purpose, 24 h after treatment, mitochondrial morphology was analyzed using CLSM following MitoTracker Orange staining. 3D reconstruction was performed to quantify mitochondrial number, volume, surface area, branching, junctions, endpoints, branch length, diameter, and sphericity. In parallel, neuronal viability, CMP depolarization, CtROS, MtROS, and intracellular ATP content were evaluated. Mdivi-1 intervention was designed as a mechanistic validation experiment to determine whether PBM-mediated restoration of mitochondrial structure and function depends on DRP-1-regulated mitochondrial dynamics. The attenuation or loss of PBM-induced improvements in mitochondrial morphology, bioenergetics, oxidative stress, and neuronal survival in the presence of Mdivi-1 was interpreted as evidence supporting the involvement of mitochondrial network remodeling in the cellular response to PBM.

### 2.1. Fluorescence Microscopy of Mitochondrial Morphology of Neurons After Aβ- and Light-Treatment

**Figure 1:**
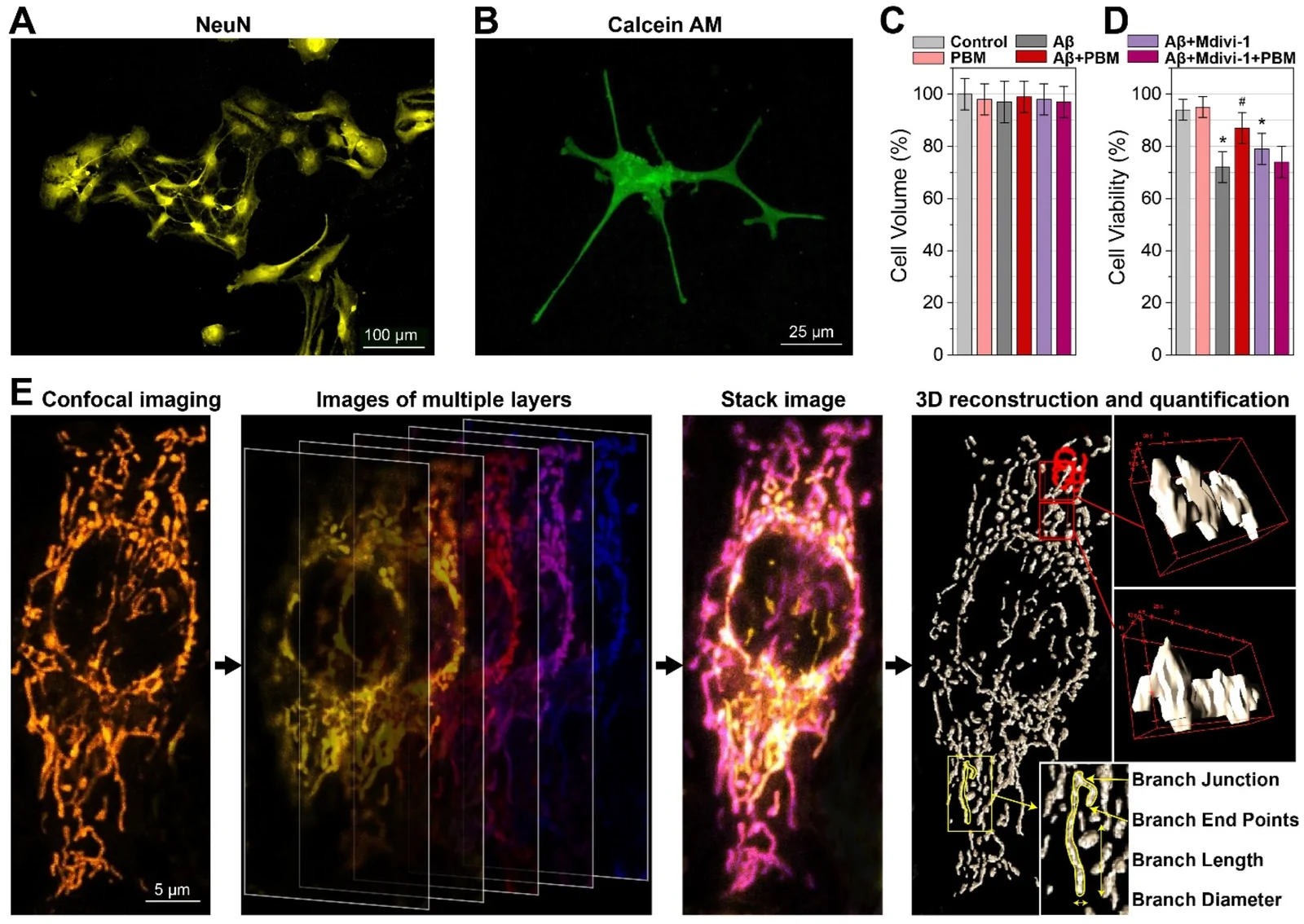
Morphological and functional characterization of primary hippocampal neurons and mitochondria after Aβ treatment and 808 nm NIR-PBM. (**A**) Epifluorescence microscopy image of primary mouse hippocampal neurons stained with neuron-selective protein antibody NeuN. (**B**) The estimation of neuron cell volume using Calcein AM epifluorescence. (**C**) Volume and (**D**) viability of neurons after Aβ treatment and PBM, where data are presented as M ± SD (*n* = 6), with * *p* < 0.05 indicating a statistically significant difference against the control and ^#^ *p* < 0.05 indicating a statistically significant difference between the Aβ group and Aβ + PBM group. (**E**) 3D mitochondrial analysis in a healthy neuron using fluorescence confocal microscopy, where representative images show mitochondria labeled with MitoTracker emitting orange light, including time-lapse z-slices, stack image projection, 3D reconstruction, and quantification of mitochondrial network morphology.

Fluorescence microscopy imaging was used for detailed quantification of the network parameters. Fluorescence microscopy imaging can allow for precise subcellular localization and quantification of complex physical and biological systems [74,75,76]. It enables high-resolution imaging of mitochondrial network in live cells, but also allows for a precise calculation of mitochondrial parameters [9,77]. Figure 1A shows the representative epifluorescence microscopy image of primary mouse hippocampal neurons specifically stained with neuron-selective protein antibody NeuN [78]. The cell volume was estimated using Calcein AM epifluorescence imaging before analyzing mitochondrial morphology (Figure 1B). Quantitative analysis revealed that no significant difference in cell volume was observed in neurons after Aβ loading and light exposure compared to intact neurons (Figure 1C), indicating that subsequent alterations in mitochondrial morphology were not associated with cell size changes.

The exposure of neurons to Aβ for 24 h induced reproducible cellular stress while preserving sufficient cell viability for morphological analyses [26]. Consistent with previous observations [26], Aβ exposure reduced neuronal viability to 72% of control values. PBM treatment significantly improved survival of Aβ-treated neurons, increasing viability to 87% (Figure 1D). Note that we used the cell counting kit (CCK) test to identify alive cells (Methods, Supplementary Materials) without identifying a ratio between necrotic and apoptotic cells.

To determine whether mitochondrial dynamics contribute to PBM-mediated neuroprotection, additional cell groups were treated with the mitochondrial dynamics inhibitor Mdivi-1. Mdivi-1 alone was found to partially improve neuronal viability in Aβ-exposed cultures to 78%, whereas the combination of PBM and Mdivi-1 produced only a modest increase to 80%, markedly lower than that observed after PBM alone (Figure 1D).

**Figure 2:**
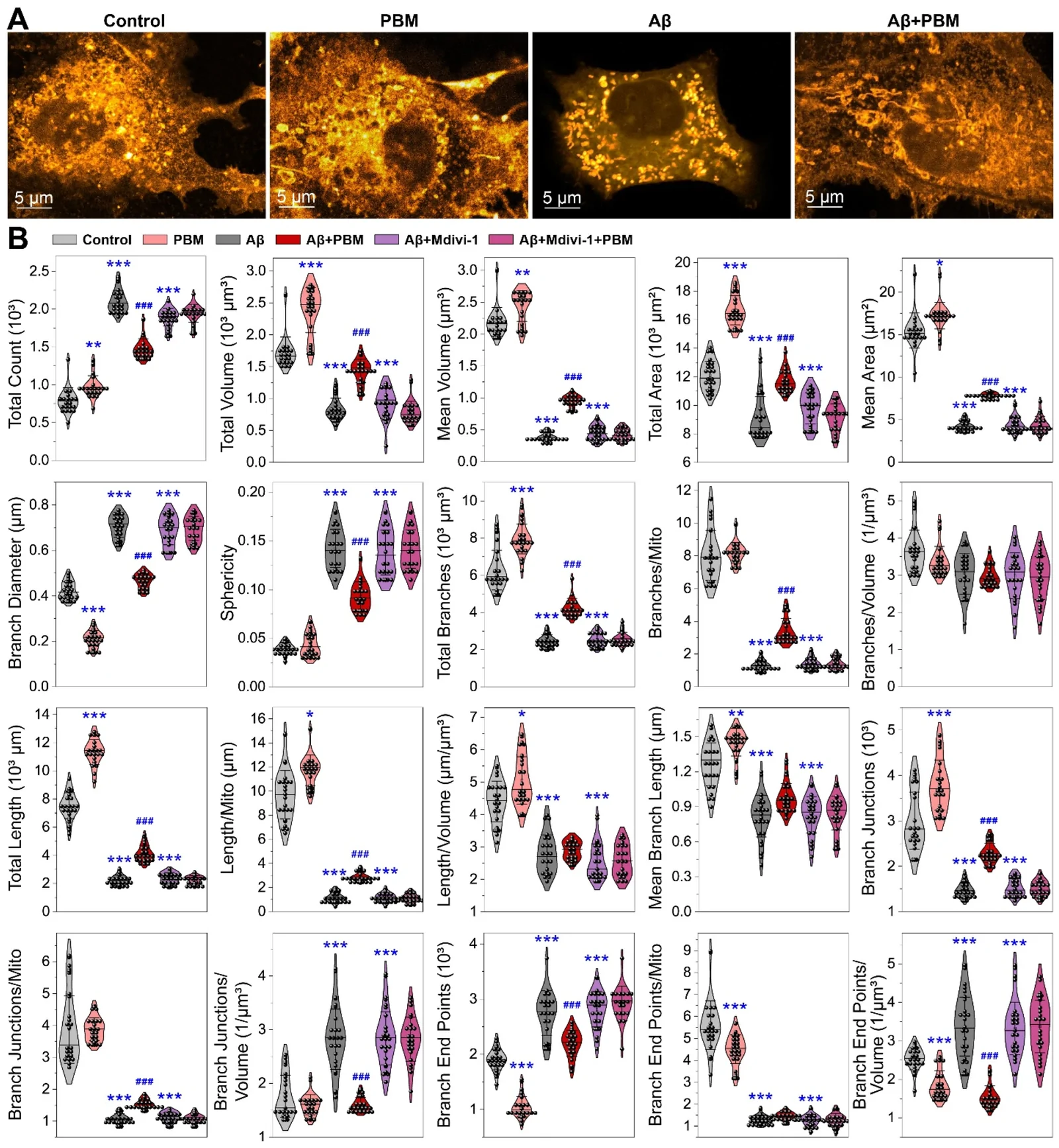
The influence of PBM with 808 nm laser (5 min, 100 mW/cm^2^, 30 J/cm^2^) on mitochondria network in healthy and Aβ-exposed neurons. (**A**) Confocal images of neurons of four groups (intact control, healthy cells 24 h after finishing PBM, cells Aβ-exposed for 24 h, and cells Aβ-exposed for 24 h immediately after PBM) stained with MitoTracker. (**B**) 3D quantification of mitochondrial parameters, including count, volume, surface area, branches, length, junctions, endpoints, sphericity, and diameter. Data are presented as M ± SD (*n* = 20), with * *p* < 0.05, ** *p* < 0.005, *** *p* < 0.001 indicating statistically significant differences against the control, and ^###^ *p* < 0.001 indicating a statistically significant difference between the Aβ group and Aβ + PBM group.

Mitochondrial network was assessed through fluorescence of MitoTracker Orange, which is known to specifically stain functionally active mitochondria [9,79]. Figure 1E represents a raw 2D CLSM image, a 3D reconstruction procedure, and structural components of mitochondria in a neuron. The operational capacity of mitochondrial network hinges upon its architectural organization and interconnections, aspects amenable to assessment via 3D confocal visualization [9]. Through scrutinizing the morphology of delineated mitochondrial network, we undertook the analysis of 20 parameters. Nine of these parameters were measured using confocal microscopy, whereas the remaining ones were subjected to quantification. This methodological strategy holds significance for acquiring more accurate data for each cell group. The parameters measured by the instrument encompass total count, total volume, surface area, branch diameter, sphericity, branch length, number of branches, junctions, and endpoints. Representative images (Figure 2A) and quantitative analysis (Figure 2B) reveal distinctive morphological alterations in mitochondrial network in control intact neurons and those 24 h after PBM, along with the cells 24 h after Aβ exposure without or with 808 nm PBM (the Aβ + PBM group).

In our experiments, the total number in the 3D morphological analysis encompassed all mitochondrial objects identified within a cell, along with their total surface area and volume (Figure 2B). This analysis computes both total and average surface area and volume, crucial for gaining more precise insights into the mitochondrial network morphology of each cell group. PBM applied to control neurons increased the total number of mitochondrial objects from 788 to 953 (+21%), total volume from 1672 to 2477 μm^3^ (+48%), average mitochondrion volume from 2.16 to 2.55 μm^3^ (+18%), total surface area from 11,944 to 16,439 μm^2^ (+38%), and average mitochondrion surface area from 15.18 to 17.30 μm^2^ (+14%). Hence, the application of PBM to healthy control neurons promotes mitochondrial biogenesis and morphological remodeling. Aβ-exposed neurons showed an increased total number to 2078 (+164%) and a decreased total volume to 798 μm^3^ (−52%), average mitochondrion volume to 0.40 μm^3^ (−82%), total surface area to 8444 μm^2^ (−29%), and average surface area to 4.19 μm^2^ (−72%). A decline in volume and surface area in Aβ-exposed neurons, along with an increased number of objects within mitochondrial network, suggests that pathological fragmentation is occurring. PBM of Aβ-exposed neurons decreased the total number of mitochondria to 1462 (−79%) but did not return to the normal number. It also increased the total volume of mitochondria to 1483 μm^3^ (+41%) and the average mitochondrion volume to 0.98 μm^3^ (+27%). The total surface area was restored to 11,549 μm^3^ (+26%) and the average surface area returned to the near-control value of 7.94 μm^2^ (+25%). The decreased number of mitochondria objects and the increased volume and surface area of mitochondrial network indicate that light can reduce Aβ-induced mitochondrial fragmentation.

Measuring the number of branches, mitochondrial length in combination with junctions and endpoints can reveal the degree of network fragmentation. Mitochondrial branch connections (junctions) characterize the connectivity of mitochondrial branches and determine the efficiency of mitochondrial network [80,81]. We assessed the branching and connectivity of mitochondrial network, using such parameters as branch, total branch length, branch junctions, and endpoints (Figure 2B). These parameters determine the functions of mitochondria and can also indicate the cell adaptation upon stress, the response to changes in bioenergetics, and during pathology [80,81].

Branching in mitochondrial networks was measured as total number of branches, branches per mitochondrion, and branches per volume (μm^−3^). After PBM of healthy neurons, the total number of branches increased from 5948 to 7853 (+32%), whereas the number of branches per mitochondrion of ~8 and per μm^3^ of ~3.5 did not change significantly. In Aβ-exposed neurons, the total number of branches was decreased to 2486 (−58%), and branches per mitochondrion were reduced to 1.28 (−84%). PBM of Aβ-exposed neurons increased the total number of branches to 4162 (+28%) and the number of branches per mitochondrion to 3.14 (+23%). The branches per volume were not changed significantly in all groups of cells.

Mitochondrial length was measured as total branch length, total branch length per mitochondrion, total branch length per volume, and mean branch length. After PBM, there were increased total branch length from 7524 to 11,340 µm (+51%), total branch length per mitochondrion from 9.7 µm to 11.7 µm (+21%), and mean branch length from 1.26 µm to 1.45 µm (+15%). Aβ-exposed neurons showed decreased total branch length to 2274 µm (−70%), total branch length per mitochondrion to 1.18 µm (−88%), and mean branch length to 0.82 µm (−35%). After PBM of Aβ-exposed cells, the total mitochondrial length increased to 4162 μm (+25%), and the total branch length per mitochondrion increased to 2.92 μm (+18%), while the total branch length per volume and mean branch length did not significantly change.

Mitochondrial branch connections are measured as total number of branch junctions, average branch junctions per mitochondrion, and branch junctions per volume. After PBM of healthy neurons (Figure 2B), we observed an increased total number of branch junctions from 3006 to 3786 (+26%), while the average branch junctions per mitochondrion of ~4 and branch junctions per μm^3^ of ~1.7 did not change significantly. In Aβ-exposed neurons, the total number of branch junctions decreased to 1480 (−51%), and the number of branch junctions per mitochondrion reduced to 1.1 (−72%), while the branch junctions per μm^3^ increased to 2.8 (+54%). PBM of Aβ-exposed cells restored the total number of branch junctions in Aβ-exposed neurons to 2295 (+27%); the branch junctions per mitochondrion increased to 1.57 (+12%), and the branch junctions per μm^3^ decreased to 1.6 (−65%).

Mitochondrial branch endpoints were measured as total number of branch endpoints, average number of branch endpoints per mitochondrion, and branch endpoints per volume. PBM in healthy neurons decreased the total branch endpoints from 1903 to 1047 (−45%), the average number of branch endpoints per mitochondrion from 5.6 to 4.5 (−19%), and the branch endpoints per μm^3^ from 2.53 to 1.86 (−26%). In Aβ-exposed neurons, the total branch endpoints increased to 2747 (+44%), the average branch endpoints per mitochondrion decreased to 1.3 (−77%), and the branch endpoints per μm^3^ increased to 3.37 (+33%). PBM of Aβ-exposed cells decreased the total branch endpoints to 2199 (−29%) and the branch endpoints per μm^3^ to 1.56 (−71%), while the average branch endpoints per mitochondrion did not significantly change.

Therefore, PBM of Aβ-exposed cells decreased the number of mitochondria objects and increased the volume, surface area, branch amount and length of mitochondrial network. This indicates that light can induce mitochondrial fusion and reduce Aβ-induced mitochondrial fragmentation. Moreover, the elongated mitochondria can be considered as an activation of mitochondrial biogenesis after light exposure. The restoration of mitochondrial branch connections is thought to improve the functioning of mitochondrial network [80,81].

A common cause of neuronal cell death is regarded as impaired mitochondrial membrane permeability, leading to mitochondrial swelling and release of CCO that activates apoptosis [81,82,83]. Alterations in cellular and mitochondrial ion transport mechanisms are pivotal in matrix swelling development [84]. Mitochondrial diameter and sphericity are parameters indicating mitochondrial network abnormalities linked to swelling. Under standard cell incubation conditions, the mean branch diameter was 0.42 µm, and the sphericity was 0.04 (Figure 2B). PBM of control cells decreased the mitochondrial diameter to 0.22 µm (−48%), without altering sphericity. The incubation of neurons with Aβ resulted in an increased diameter to 0.72 μm (+71%) and an enlarged sphericity to 0.14 (+258%). PBM of Aβ-exposed neurons restored the diameter to almost the control value of 0.47 µm (−57%) and sphericity to 0.09 (−128%). The obtained results may indicate that Aβ disrupts the balance of ions in mitochondria, swelling the matrix, which leads to cell death. Mitochondrial swelling is usually an irreversible process in which mitochondria are either utilized by lysosomes or activate apoptosis [85,86]. Our results showed that mitochondrial swelling can be partially reversible after PBM.

To determine whether mitochondrial dynamics is required for these PBM-induced structural effects, neurons were treated with the DRP-1 inhibitor Mdivi-1 at 50 μM. Mdivi-1 alone failed to improve mitochondrial morphology in Aβ-treated neurons (no statistical difference compared to the Aβ group). In the Aβ + Mdivi-1 group, mitochondrial count of 1909, total volume of 982 μm^3^, surface area of 10,121 μm^2^, branch number of 2523, branch length of 2410 μm, junction number of 1538, mitochondrial diameter of 0.7 μm, and sphericity of 0.13 remained close to the values observed in the Aβ group. Also, no significant structural improvements were observed in the Aβ + Mdivi-1 + PBM group: mitochondrial count of 1952, total volume of 758 μm^3^, surface area of 9480 μm^2^, branch number of 2593, branch length of 2296 μm, junction number of 1517, mitochondrial diameter of 0.7 μm, and sphericity of 0.14 remained statistically unchanged compared to the Aβ-treated neurons. The main presented quantitative data on mitochondrial structure are given in Table 1. Also, the Mdivi-1 and Mdivi-1 + PBM groups were compared for core mitochondrial network morphology parameters. Mdivi-1 induced no statistically significant changes in mitochondrial volume, branching, diameter or sphericity relative to the control and PBM group, respectively (Figure S1A, Supplementary Materials).

### 2.2. Impact of Aβ and PBM on Neuronal Membrane Potential, ROS, and ATP Production

**Figure 3:**
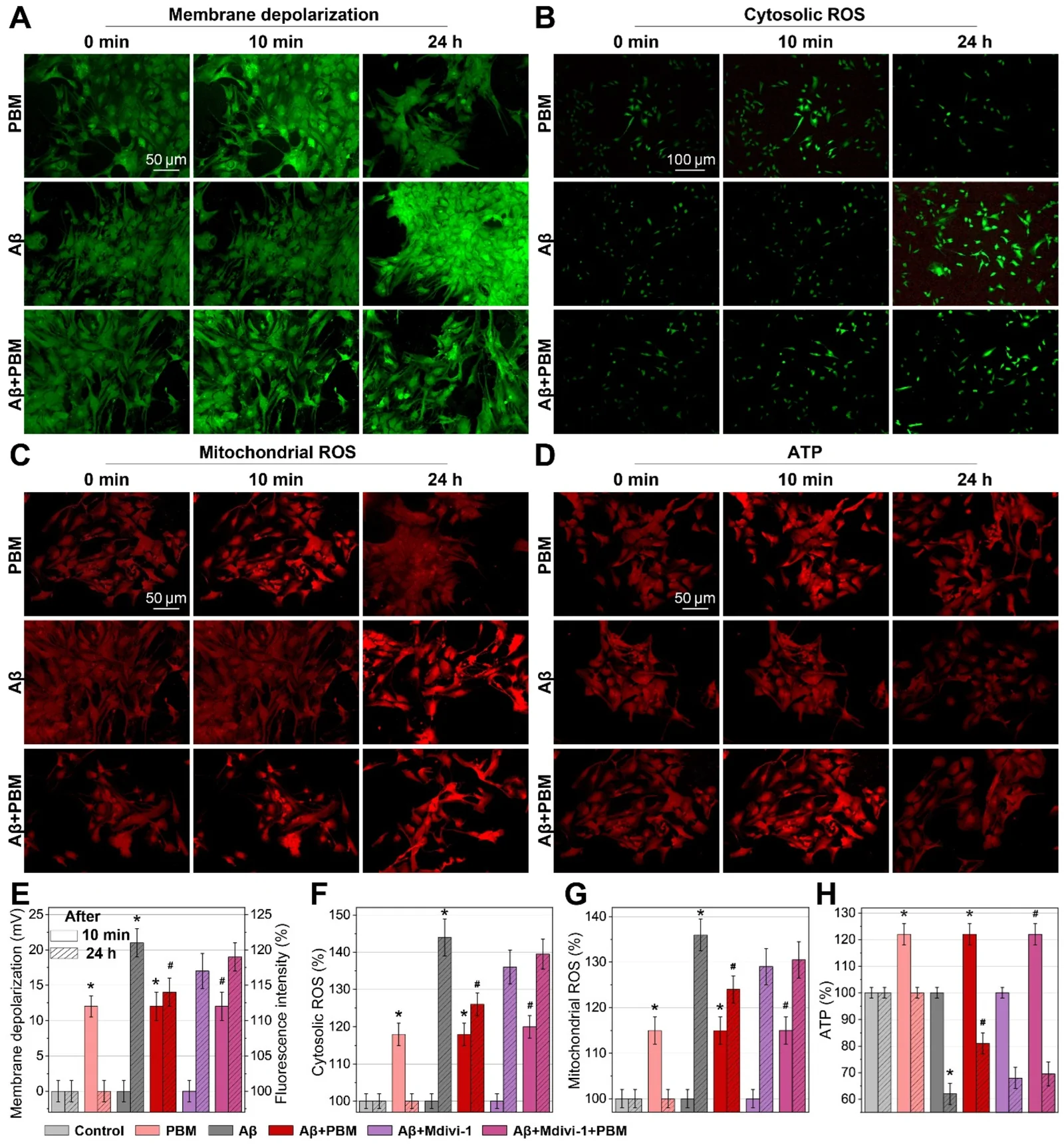
Impact of Aβ and 808 nm PBM (5 min, 100 mW/cm^2^, 30 J/cm^2^) on neuronal CMP, ROS, and ATP production measured after 10 min and 24 h using epifluorescence microscopy of neurons stained with fluorescent probes. Representative fluorescence images and histograms of changes in (**A**,**E**) membrane depolarization, (**B**,**F**) cytosolic and (**C**,**G**) mitochondrial ROS generation, and (**D**,**H**) ATP production following 10 min and 24 h after PBM, Aβ exposure, and Aβ + PBM treatments. Data are presented as M ± SD, with * *p* < 0.05 indicating statistically significant differences against the control and ^#^ *p* < 0.05 indicating a statistically significant difference against the Aβ group.

To determine whether the structural remodeling of the mitochondrial network was accompanied by functional recovery, we quantified early (after 10 min) and late (after 24 h) changes in MtMP, CtROS/MtROS generation, and ATP production using epifluorescence microscopy (representative images are presented in Figure 3A–D).

For assessing the influence of Aβ and PBM on CMP depolarization, we utilized the CMP-sensitive probe DiBAC4(3). The fluorescence intensity of this probe can be quantified, enabling the evaluation of changes in CMP relative to the resting CMP (1% of intensity increase is linearly proportional to ~1 mV of membrane depolarization) [55]. In Figure 3A,E, the CMP profile showed that PBM increased CMP depolarization by 12 mV after 10 min (as shown in Ref. [55], this effect reverted to the initial level within a few minutes). Adding Aβ to neurons had no impact after 10 min, yet a significant 21 mV increase in CMP was observed after 24 h. Importantly, Figure 3A,E shows that PBM reduced the CMP depolarization in Aβ-exposed neurons after 24 h, normalizing CMP by 7 mV (i.e., by 33%).

Simultaneously, PBM of healthy neurons increased CtROS by 18% (Figure 3B,F) and MtROS by 15% (Figure 3C,G) after 10 min, and these concentrations returned to the initial values within 24 h. Such a transient increase is within the physiological range for cells. In contrast, culturing neurons with Aβ did not alter ROS concentrations after 10 min but caused a substantial increase in CtROS by 44% and MtROS by 36% after 24 h. PBM mitigated these effects in Aβ-exposed neurons, decreasing CtROS by 18% and MtROS by 12% after 24 h (Figure 3B,C).

PBM enhanced ATP production by 22% after 10 min, with the levels returning to baseline within 24 h. Culturing neurons with Aβ did not affect ATP concentration after 10 min but led to a 38% decrease after 24 h. PBM normalized ATP production in Aβ-exposed cells after 24 h by 19% (Figure 3D,H).

Similar immediate responses (after 10 min) were observed in the Aβ + PBM and Aβ + PBM + Mdivi-1 groups, indicating that DRP-1 inhibition did not prevent the initial PBM-induced activation of mitochondria. Treatment with Mdivi-1 alone partially attenuated Aβ-induced dysfunction. In the Aβ + Mdivi-1 group after 24 h, CMP was depolarized by 17 mV, ATP level increased to 68%, and both CtROS and MtROS concentrations were moderately reduced to 136% and 129%, respectively (Figure 3E–H, compared to the Aβ group). Neuronal viability also improved to 79% (Figure 1D). Also, the Mdivi-1 and Mdivi-1 + PBM groups were compared for intracellular ATP, ROS, and viability (Figure S1B,C, Supplementary Materials). Mdivi-1 alone induced no statistically significant changes in viability, ATP levels, or ROS generation relative to the control.

Importantly, the inhibition of DRP-1 largely abolished the long-term protective effects of PBM. Although the early PBM-induced increases in ATP production and transient ROS signaling remained detectable after 10 min in the presence of Mdivi-1, these acute responses did not translate into sustained functional recovery. At 24 h, neurons treated with Aβ + Mdivi-1 + PBM exhibited CMP depolarization of 19 mV, ATP concentration of only 69%, CtROS concentration of 139%, and MtROS concentration of 130% (Figure 3E–H). Neuronal viability reached only 74% (Figure 1D), closely resembling the values observed in the Aβ group and showing statistically non-significant changes compared to the Aβ + Mdivi-1 group. The main presented quantitative data on cellular pathways after the influence of Aβ and/or PBM are given in Table 1 for correlative analysis with those on mitochondrial structure.

**Figure 4:**
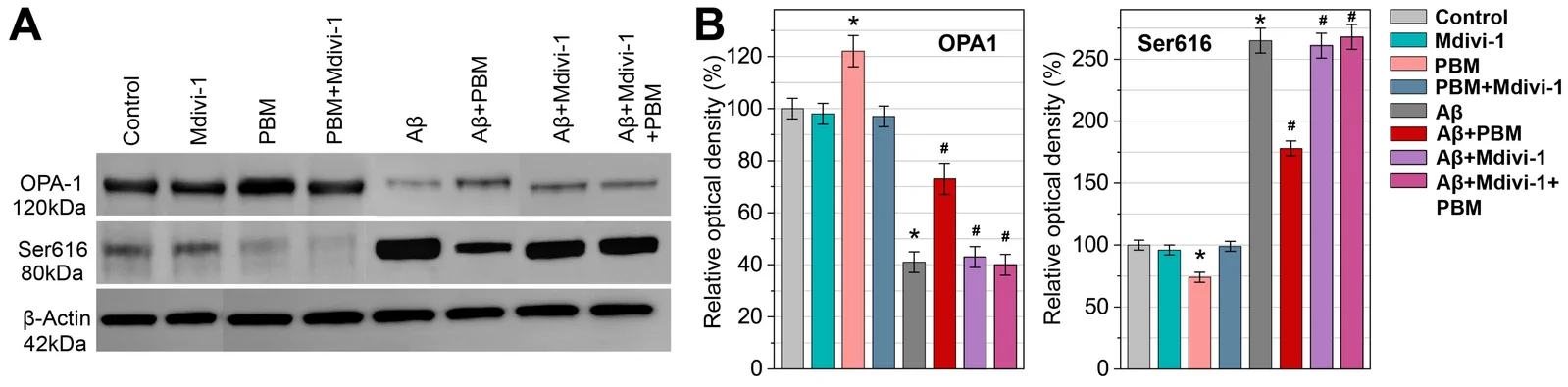
Immunoblot of OPA-1, phosphorylated DRP-1 (Ser616), and β-actin loading control across all experimental groups after 24 h. (**A**) Lanes of sequential treatment groups: control, Mdivi-1, PBM, PBM + Mdivi-1, Aβ, Aβ + PBM, Aβ + Mdivi-1, Aβ + PBM + Mdivi-1. (**B**) Immunoblot band densitometry quantification, where the blot signal intensities were normalized to β-Actin to correct for unequal protein loading across samples. Data are presented as M ± SD, with * *p* < 0.05 indicating statistically significant differences against the control and ^#^ *p* < 0.05 indicating a statistically significant difference against the Aβ group.

To mechanistically link the early PBM-triggered ATP elevation and late mitochondrial network remodeling, Western blot quantification of fusion protein OPA-1 and fission-activating phosphorylated DRP-1 (p-DRP, Ser616) was performed for the 24 h experimental endpoint, with representative raw blots in Figure S2 and consolidated summary blots in Figure 4. Western blot analysis of OPA-1 and p-DRP revealed distinct DRP-1-dependent regulatory effects of PBM at the 24 h timepoint. In healthy neurons, Mdivi-1 treatment alone induced no significant shifts in OPA-1 or p-DRP-1 abundance relative to the untreated control. PBM exposure 22% upregulated OPA-1 expression and 25% reduced basal p-DRP-1 expression in intact neurons; the co-administration of Mdivi-1 fully reversed both light-induced changes. Following 24 h Aβ oligomer toxicity, neurons exhibited 59% OPA1 downregulation and 163% elevated p-DRP-1 expression, consistent with excessive pathological mitochondrial fission. PBM treatment partially restored Aβ-triggered OPA-1 abundance by 32% and p-DRP-1 expression by 86%. Strikingly, pre-incubation with Mdivi-1 completely abolished all PBM-driven modulations of OPA-1 and p-DRP-1 in Aβ-treated neurons, confirming functional DRP-1 activity required for sustained PBM-mediated mitochondrial network remodeling. No significant changes in OPA1 or p-DRP-1 expression were detected at the early 10 min post-PBM timepoint, separating rapid DRP-1-independent metabolic stimulation from delayed DRP-1-dependent protein regulatory effects.

## 3. Discussion

The role of mitochondrial network remodeling in NIR-PBM-mediated neuroprotection in primary mouse hippocampal neurons exposed to Aβ was investigated. Aβ caused swelling and fragmentation of mitochondria, decreasing their volume and area as well as disrupting the connectivity of mitochondrial network. Morphological changes in mitochondrial network after PBM, such as restored total number of mitochondria, reduced degree of their swelling, and increased length and connectivity indicated that PBM can reduce the pathological fragmentation of mitochondria induced by Aβ and improve mitochondrial integrity and biogenesis [68,80,81]. Moreover, the application of PBM to healthy neurons promoted mitochondrial biogenesis and morphological remodeling (enhancing mitochondrial mass). The mitochondrial structural alterations in neurons exposed to Aβ were accompanied by elevated ROS production, ATP depletion, membrane depolarization, and reduced neuronal viability. PBM significantly reversed these abnormalities, restoring mitochondrial network integrity and partially normalizing cellular bioenergetics and redox homeostasis.

Neurons were additionally treated with the DRP-1 inhibitor Mdivi-1 [87], showing that the mitochondrial morphological profiles of the Aβ + PBM + Mdivi-1 and Aβ + Mdivi-1 groups were largely indistinguishable. At the same time, the treatment with Mdivi-1 alone partially attenuated Aβ-induced dysfunction: ATP concentration increased, and both CtROS and MtROS concentrations were moderately reduced. Neuronal viability was also improved, indicating that even partial suppression of excessive mitochondrial fission somewhat alleviated Aβ-induced toxicity. On the other hand, the inhibition of DRP-1 largely abolished the long-term protective effects of PBM. It should be noted that, while high concentrations of Mdivi-1 can exert mild complex I suppression in certain cell types, our readouts of basal bioenergetics and mitochondrial structure confirm that 50 μM Mdivi-1 does not induce measurable respiratory dysfunction or morphological damage in healthy hippocampal neurons under our culture conditions. These findings demonstrate that PBM can elicit a mitochondrial response that is independent of DRP-1 activity; nevertheless, sustained improvements in mitochondrial function, redox balance, ATP production, and neuronal survival require intact mitochondrial network remodeling.

The data on the negative influence of Aβ on the biochemical pathways related to mitochondria are consistent with the reported information. Aβ triggers cell membrane depolarization via disrupted ion homeostasis, binding to N-methyl-D-aspartate receptors, or it forms membrane pores to boost Ca^2+^ influx, activating Ca^2+^-dependent K^+^ channels and K^+^ efflux [88]. Aβ also disrupts the membrane bilayer [26]. These events impair Na^+^/K^+^-ATPase, critical for CMP maintenance, causing Na^+^ buildup and K^+^ loss, upregulating Cl^−^ channels (e.g., TMEM16A) to increase Cl^−^ entry [89]. This ion imbalance drives ROS overproduction: excess cytosolic Ca^2+^ floods mitochondria, overactivating the ETC and causing electron leakage, which generates MtROS [89]. Meanwhile, the depolarization and Na^+^/K^+^-ATPase impairment increase cytosolic stress, activating NADPH oxidases (NOX), which is a key source of CtROS in Aβ-induced neurotoxicity. Aβ also reduces ATP, inhibiting a major ATP consumer Na^+^/K^+^-ATPase initially spares ATP, but MtROS damages ETC components (e.g., complex IV), slowing ATP synthesis [90]. Additionally, Ca^2+^ overload triggers mitochondrial permeability transition pore (mPTP) opening, further disrupting ETC function and depleting ATP stores [91]. Consequently, the balance of mitochondrial fission and fusion collapses due to the accumulation of damaged mitochondria, leading to overexpression of both fission and fusion genes to restore homeostasis: splitting more damaged organelles and merging remaining healthy ones. This creates a hyperactive but ineffective stress response. Finally, mitochondrial fragmentation impairs cellular bioenergetics, being associated with neuronal apoptosis during the progression of neurological diseases [92,93,94]. Therefore, AD brains have damaged mitochondria caused by Aβ toxicity, neurofibrillary tangles, and oxidative stress.

We suggest that PBM boosts mitochondrial function through instant light-induced CCO activation, ROS generation, Ca^2+^ signaling, and ATP production. These responses appeared the same in either normal or Aβ-exposed neurons. It is known for intact cells that the initial phase involves red/NIR light interacting with cells via CCO, located in mitochondria [41,42]. Following photoactivation, CCO elevates MtMP, consequently affecting various intracellular pathways [43,44,45,46], through ROS production [41,42], ATP synthesis [43,48,49], and caspase family members activation [51,52]. CCO activation enhances mitochondrial respiration and ATP generation while lowering excessive ROS production; these downstream effects are known to modulate core mitochondrial fission and fusion machinery, including DRP-1, MFN-1/2, and OPA-1 [44,46,95,96,97,98].

The results showed that the acute 10 min PBM-driven elevation in ATP occurs independently of DRP-1 function, as this transient bioenergetic boost persists even when DRP-1 is pharmacologically inhibited with Mdivi-1. This immediate photochemical effect may stem from direct light-mediated CCO activation, rapidly boosting the ETC efficiency without requiring transcriptional or post-translational shifts in mitochondrial fission/fusion proteins. However, this short-lived bioenergetic enhancement alone cannot sustain long-term mitochondrial network integrity after 24 h Aβ toxicity. As validated via OPA-1 and p-DRP-1 immunoblot data, sustained restoration of balanced mitochondrial dynamics requires downstream DRP-1-dependent signaling: the initial ATP surge supplies the bioenergetic substrate to support regulated post-translational modification and balanced turnover of DRP-1 and fusion protein OPA-1 over extended culture. When DRP-1 activity is blocked by Mdivi-1, the early PBM metabolic boost fails to counteract Aβ-induced DRP-1 hyperphosphorylation and OPA-1 degradation over 24 h, eliminating the delayed structural rescue of fragmented mitochondrial networks. This two-phase mechanistic model differentiates rapid, DRP-1-independent respiratory stimulation from delayed, DRP-1-required mitochondrial structural remodeling, resolving the disconnect between short-term metabolic responses and long-term morphological protection observed in our model. Therefore, the inability of PBM to confer long-term neuroprotection in the presence of a DRP-1 inhibitor Mdivi-1 suggests that PBM-mediated restoration of mitochondrial networks does not act solely by promoting fusion; instead, it requires functional DRP-1 to reverse Aβ-induced excessive fission. When DRP-1 is inhibited, PBM does not remodel fragmented mitochondria to re-establish interconnected networks, even though the fusion machinery is not inhibited. This suppression of PBM protective effects demonstrates that balanced fission–fusion activity is a prerequisite for PBM to normalize network integrity (via a complex route of remodeling [69,81,83]). Hence, PBM triggers early metabolic signaling, but sustained morphological and functional recovery in Aβ-challenged neurons depends on unblocked mitochondrial fission–fusion machinery coordinated by proteins responsible for mitochondrial remodeling.

The participation of CCO–NO complexes, suppressing ETC flux, should also be considered. A major conserved mechanism of red/NIR-PBM is photon-induced photodissociation of inhibitory CCO–NO complexes, relieving NO-mediated respiratory chain suppression and restoring CCO catalytic activity [47]. Importantly, our in vitro monoculture model lacks endogenous NO sources present in clinical AD brains: activated microglia, astrocytes, and endothelial cells produce high levels of NO under neuroinflammatory conditions, creating abundant CCO–NO complexes that can be targeted by PBM therapy in patients. In our neuron-only culture, NO concentrations are minimal, so we suggest that the observed mitochondrial rescue effects are driven primarily by direct light stimulation of unoccupied CCO and downstream modulation of mitochondrial fission/fusion dynamics via DRP-1. In clinical AD, PBM would exert dual beneficial actions: (i) direct relief of NO-mediated CCO inhibition via photodissociation of CCO–NO complexes and (ii) restoration of fragmented mitochondrial networks to normalize neuronal bioenergetics.

Additionally, red/NIR light can depolarize CMP, leading to the short-term activation of Ca^2+^ channels and the ER [53,54,55,56]. At the same time, we found that light-induced signaling partially reversed the long-term Aβ-related stress in AD model neurons; namely, alleviating ROS and membrane depolarization, as well as increasing ATP production. Restoring mitochondrial homeostasis, PBM apparently reduces mitochondrial damage and improves MtMP. Thus, PBM acts as a stress reducer, restoring ETC function and global homeostasis. With partially normalized mitochondria, the cell loses the need for hyperactive fission or fusion; their activity drops toward a physiological level. All this may explain the subsequent light-induced decrease in mitochondrial fragmentation after some hours after PBM application. Indeed, it is known that red/NIR-PBM can upsurge mitochondrial oxidative stress alongside the regulation of antioxidant genes, facilitating cellular proliferation and enhancing survival rates for one–two days [25,29].

Thus, since the recovery of these pathways is related to energy metabolism, inflammation regulation, protection from apoptosis, and DNA repair, this suggests that PBM of AD mice can provide neuroprotection and improve cognitive functions via a substantial role of restored mitochondrial structure and energy metabolism. This agrees with the fact that red/NIR-PBM is known to enhance spatial memory, motor functionality, and cognitive capabilities [17,22,26,27,28,31,99] as a result of the alleviated Aβ plaque burden [18,19,20,21,22,23,24,25,26,27,28], neuronal damage [24,25,26,27,28,29], and microgliosis [20,21,22,27,29]. At the same time, PBM can restore mitochondrial functioning via normalizing MtMP in neurons [55,63] and other cells [41,42,43,44,45,46,64,65,66]. NIR light was also reported to instigate changes in mitochondrial fission and fusion during wound healing [69]. As for AD, PBM can modulate mitochondrial energy metabolism and reduce neurological damage in AD mice [28]. Hence, by targeting mitochondrial function via transcriptional normalization, structural repair, and functional improvement, NIR-PBM represents a promising therapeutic strategy for AD.

This in vitro study has several key limitations restricting broad interpretation. First of all, only isolated mouse hippocampal neurons were used, lacking astrocytes, microglia and vascular cells. Hence, native neuron–glia crosstalk controlling Aβ clearance, inflammation and mitochondrial balance was not recapitulated. Microglia activated by Aβ release pro-inflammatory cytokines (TNF-α, IL-1β) that further shift mitochondrial dynamics toward excessive fission in neurons, while reactive astrocytes lose homeostatic support functions including lactate bioenergetic shuttling and antioxidant supply to neurons [100]. Furthermore, while this study focuses on hippocampal neurons, multiple interconnected brain regions exhibit pathological changes at prodromal and early symptomatic AD stages. The entorhinal cortex bears the earliest tau neurofibrillary tangles, acting as the primary starting point of pathological protein spreading. Additional vulnerable regions include the perirhinal and parahippocampal cortices, medial prefrontal cortex, posterior cingulate cortex, inferior temporal gyrus, and basal forebrain cholinergic nuclei, all critical for memory encoding, retrieval, and self-referential cognition [101,102]. Widespread mitochondrial dysfunction and Aβ accumulation arise across these interconnected limbic and association cortices prior to global brain atrophy, highlighting that hippocampal impairment represents only one component of widespread early AD neurodegeneration [12].

We utilized an acute 24 h oligomeric Aβ_1–42_ (10 μM) treatment model to rapidly induce robust and quantifiable mitochondrial structural and functional impairments. This acute toxic paradigm differs from clinical AD, where soluble Aβ oligomers accumulate slowly over 10–30 years. Chronic low-level Aβ burden induces sustained mitophagy impairment, epigenetic downregulation of mitochondrial biogenesis genes, and permanent impairment of ER–mitochondrial calcium crosstalk, the pathological signatures absent after only 24 h of Aβ treatment. Moreover, the 10 μM Aβ concentration used herein is substantially higher than picomolar soluble Aβ oligomer concentrations measured in cerebrospinal fluid and interstitial fluid of AD patients [73]. Supraphysiological Aβ concentrations amplify calcium influx, ETC leakage, and ROS overproduction, accelerating mitochondrial swelling and fragmentation. At physiological picomolar Aβ concentrations in vivo, ROS generation occurs much more gradually, meaning mild mitochondrial dysfunction. In our acute setting, PBM fully reverses most Aβ-induced mitochondrial fragmentation and bioenergetic deficits. Moreover, our experimental design isolates only Aβ oligomer-induced mitochondrial damage to dissect the specific mitochondrial remodeling mechanisms of 808 nm PBM without confounding tau toxicity [15], which represents another limitation for mechanistic deconvolution. In the case of PBM therapy in chronic clinical AD, accumulated irreversible mitochondrial damage may only partially rescue neurons, requiring repeated PBM dosing regimens to sustain gradual mitochondrial network remodeling. Nevertheless, our core mechanistic conclusion, that PBM relies on DRP-1-dependent mitochondrial dynamics to rescue damaged mitochondrial networks, remains generalizable across Aβ doses, as DRP-1 hyperactivation is a conserved feature of both high-dose in vitro and low-dose chronic in vivo Aβ toxicity.

Another limitation is that all data originate from neonatal neurons in vitro; hence, transgenic AD animal in vivo experiments are missing to support clinical translation. Also, the long-term persistence of PBM-mediated mitochondrial protection against chronic Aβ damage remains unassessed. Since only one 808 nm PBM dose was examined, future research should be focused on wavelength gradient or dose–response screening to identify optimal therapeutic parameters [24,42,103]. Additionally, photochemically modulated mitochondrial biomolecular pathways were only initially studied so far. Since only mitochondrial fission/fusion proteins OPA-1 and p-DRP-1 were detected, comprehensive RNA/DNA/proteome profiling, full mitochondrial signaling pathway dissection, and quantitative inflammation marker measurements are important [26,61]. Also, mitophagy-related signaling and regulatory proteins (e.g., LC3-II, p62, colocalization with LAMP-1) were not analyzed, so this research cannot clarify whether PBM remodels damaged mitochondria via mitophagy to eliminate dysfunctional organelles [86,104]. Despite these constraints, this work provides quantitative 3D mitochondrial morphological evidence that DRP-1-dependent mitochondrial dynamics are essential for 808 nm PBM neuroprotection against acute Aβ injury, laying a foundation for follow-up multicellular, omics, longitudinal and in vivo research.

## 4. Materials and Methods

### 4.1. Isolation and Culture of Primary Mouse Hippocampal Neurons

Primary mouse hippocampal neurons were isolated from postnatal C57BL/6J mouse pups, purchased from Guangzhou Huateng Biomedical Technology, Guangzhou, China. All animal procedures were performed in strict accordance with Experimental Animal Use Regulations of Guangdong Province Department of Science and Technology (permit No. SYXK-2024-0307) and the National Institutes of Health Guidelines for the Care and Use of Laboratory Animals. The neuron isolation method is detailed in Methods, Supplementary Materials. The in vivo experimental protocols were approved by the Experimental Animal Ethics Committee of the Medical Department of Shenzhen University (approval number A202600900, issued by 13 March 2025). All animal procedures strictly complied with the Guide for the Care and Use of Laboratory Animals (National Academy Press, Washington, DC, USA, 1996). Briefly, primary hippocampal neurons were isolated from postnatal day 0 to day 1 C57BL/6J mouse pups. Euthanasia of mouse pups via the physical method was performed after anesthesia with isoflurane in a chamber, in compliance with the guidelines for neonatal rodents. The brains were collected, resuspended, and centrifuged to extract viable cells. The cells were plated at 1 × 10^6^ cells per 35 mm dish for 24 h for adhesion and then cultured for 12 days in complete culture medium for mouse hippocampal neuron cells: Eagle’s minimum essential medium/F12, 15% fetal bovine serum, 1% penicillin–streptomycin solution (VisionEstern, Shenzhen, China) at 37 °C in a 95% humidified atmosphere with 5% CO_2_ [105].

### 4.2. Alzheimer’s Disease In Vitro Model

To create an AD in vitro model, the cultured neurons were treated with soluble oligomeric Aβ_1–42_ (Thermo Fisher Scientific, Waltham, MA, USA) at a concentration of 10 μM for 24 h to induce observable cellular changes in a short timeframe during experimental modeling (the preparation and use are detailed in Ref. [26]). To mitigate the dynamic instability of soluble Aβ_1–42_ oligomers which can undergo conformational rearrangement into inert low-toxicity mature fibrils during prolonged culture incubation, we prepared fresh oligomeric Aβ_1–42_ immediately before each cell treatment, applied the peptide solution to neuronal cultures within 30 min of oligomerization, and performed parallel immunofluorescence labeling with oligomer-specific Aβ antibodies to validate sustained oligomeric species abundance at the 24 h experimental endpoint (Figure S3). After cultivation, the used medium was removed, and neurons were washed thoroughly to remove the Aβ-containing medium. Right after washing, either fresh pure medium or medium containing 10 μM Aβ_1–42_ was added to cell dishes. All dishes with cultured neurons with fresh medium added were divided into 4 groups (three dishes per group): control untreated cells, cells irradiated with 808 nm light, cells incubated with Aβ for 24 h, and Aβ-exposed cells irradiated with 808 nm light.

### 4.3. Pharmacological Inhibition of Mitochondrial Dynamics Using Mdivi-1

To investigate whether mitochondrial network remodeling contributes to the neuroprotective effects of PBM, mitochondrial reshaping (fission was pharmacologically inhibited using Mdivi-1 (Sigma-Aldrich, Merck, Darmstadt, Germany), which is a selective inhibitor of DRP-1 [87]. Primary hippocampal neurons were pre-incubated with Mdivi-1 at a final concentration of 50 μM for 1 h before the addition of oligomeric Aβ_1–42_. The inhibitor remained present in the culture medium throughout the subsequent experimental period, including Aβ exposure and PBM treatment.

### 4.4. Light Treatment

Neurons in glass bottom cell culture dishes were irradiated using an 808 nm diode laser Laserland LLLD808CW200 (Laserland, Wuhan, China) at a light power density of 100 mW/cm^2^ for 5 min (a resultant irradiation dose of 30 J/cm^2^). While being illuminated, each dish was placed on a 37 °C thermally stabilized platform for cell dishes. To minimize reflective light loss, a defocused laser beam was directed near-perpendicular to the platform surface. A calibrated optical power meter was used to measure light power directly under the cell culture dish plane under a dish filled only with pure medium to acquire the dose of the delivered light. The measured power differences because of surface reflection and measurement uncertainties can be below 4%, which is a negligible deviation that cannot alter the target dosing. Bearing in mind the biphasic biological response to PBM, these light power density (100 mW/cm^2^) and dose (30 J/cm^2^) were chosen because NIR light with such parameters is known to provide positive effects on cells and tissues, while not causing cell phototoxicity and capable of stimulating CCO activity without inducing lethal cellular stress [24,43,55,63,106,107]. Light irradiation was performed minutes after the addition of fresh medium (pure or with Aβ/Mdivi-1) described above. The temperature changes in cell culture dishes with cells in medium under illumination at the abovementioned parameters were monitored using a thermal imaging camera EVIDIR Alpha (Jenoptik, Jena, Germany), showing that the temperature of irradiated cell culture dishes did not rise during 20 min.

### 4.5. Confocal Laser Scanning Microscopy Imaging

CLSM imaging was conducted on primary neurons seeded into 35 mm glass bottom dishes for 24 h, with or without oligomeric Aβ_1–42_ and light irradiation. After removal of old medium, the cells were stained with 1 μg/mL MitoTracker Orange CM-H2TMRos (1 μg/mL, *λ_exc_* = 554 nm/*λ_em_* = 576 nm, Thermo Fisher Scientific, Waltham, MA, USA) in a fresh medium for 15 min at 37 °C [79]. The fluorescent mitochondrial probe was used for labeling live mitochondria for further reconstruction of the mitochondrial network. Following staining, neurons were washed thoroughly and placed in a fresh phenol red-free medium. CLSM imaging was performed using a confocal laser scanning microscope Nikon A1RMP+ (Nikon, Tokyo, Japan) with a 60× oil immersion objective (Nikon, Tokyo, Japan) right after labeling. In addition to planar XY images, z-stacks were acquired for 3D cell imaging. The z-size in this case was ~18 μm, and the *z*-spacing was 250 nm.

The images were processed using ImageJ 2.0.0, with raw data displayed unless specified otherwise. A full mitochondrial reconstruction was created by integrating stacked slices with ImageJ. Z-stacks of images detailed mitochondrial networks displayed in pseudo-color. Mitochondrial parameters, such as count, volume, surface area, branches, length, junctions, endpoints, sphericity, and diameter, were quantified using Mitochondrial Analyzer 3D plugin [108,109]. Thresholding and contrast optimization were automatically adjusted [77] to ensure precise network delineation and distinguish tightly packed mitochondrial components (peculiar parameters are described in Methods, Supplementary Materials). For 3D cell volume measurement, we ran the macro code in ImageJ to quantify the plasma membrane area in each frame using Calcein AM staining at 3 μg/mL (Thermo Fisher Scientific, Waltham, MA, USA). These measurements were then combined to reconstruct the 3D images representing cell volume.

### 4.6. Immunohistochemistry and Epifluorescence Microscopy

Fluorescent probes and antibodies were purchased from Thermo Fisher Scientific (Waltham, MA, USA) and used according to the manufacturer’s instructions. Fluorescent oligomeric Aβ_1–42_ (green emission, 490/515 nm) and neuron-selective protein NeuN (green emission, 506/517 nm), a CMP-sensitive probe DiBAC4(3) (green emission, 490/516 nm), CM-H2DCFDA CtROS probe (green emission, 504/525 nm), MitoSox Red MtROS probe (red emission, 396/610 nm), and BioTracker ATP probe (red emission, 590/615 nm), were applied at 2 μg/mL concentration. Immunofluorescence microscopy imaging was performed using a Nikon Eclipse Ti-U inverted fluorescence microscope (Nikon, Tokyo, Japan). The obtained epifluorescence images were analyzed using the Nikon microscope software (NIS-Elements D) to integrate emission intensity over the field of view.

### 4.7. Statistical Analysis

All data were statistically analyzed using OriginLab 2021 9.8.0.200 software by means of two-way ANOVA with Tukey’s post hoc test. Before the ANOVA test, the Shapiro–Wilk test was applied to verify the normal distribution of all morphological, physiological and viability datasets. Only normally distributed data were included for parametric statistical comparison. All results were presented as mean ± standard deviation (M ± SD), with *p* < 0.05 defined as statistically significant.

## 5. Conclusions

This study demonstrated that NIR-PBM with 808 nm laser (100 mW/cm^2^, 30 J/cm^2^) of AD in vitro model provided multitargeted neuroprotection via restoring mitochondrial structure and energy metabolism in neurons. CLSM of neurons followed by image analysis indicated that PBM itself enhanced the total number of mitochondrial objects, branches and junctions by ~25% and increased overall volume and surface area by ~38%. The incubation with Aβ caused increased mitochondrial fragmentation by 164% and diameter by 69% and decreased overall volume by 51%, surface area by 24%, and network connectivity by 48%; while PBM reversed these disruptions, decreasing the network fragmentation by 79% and diameter by 57% and increasing overall volume by 34%, surface area by 21%, and network connectivity by 24%.

To elucidate the effects of Aβ and 808 nm NIR-PBM on mitochondrial physiology in neurons, we analyzed CMP depolarization, CtROS/MtROS generation, and ATP production at 10 min and 24 h after the exposure. After 24 h, Aβ increased CMP by 21 mV, CtROS by 44%, and MtROS by 36% and decreased ATP production by 38%. PBM resulted in instant biochemical responses in both healthy and Aβ-exposed neurons. In healthy cells, light increased CtROS by 44%, MtROS by 36%, and ATP by 22% after 24 h, while depolarization was observed only during light illumination. More importantly, PBM of the Aβ-exposed neurons led to partially decreased CMP by 7%, CtROS by 18%, and MtROS by 12% and restored ATP production by 19% after 24 h.

To determine whether mitochondrial dynamics are essential for PBM-induced effects, neurons were additionally treated with the DRP-1 inhibitor Mdivi-1. PBM failed to restore mitochondrial connectivity, suppress oxidative stress, recover ATP production, or improve neuronal survival at the blockade of DRP-1. Therefore, PBM was shown to act as a stress reducer, restoring ETC function and global homeostasis. With partially normalized mitochondria, their fission and fusion activities drop toward a physiological level.

In summary, our findings establish a mechanistic link between PBM-mediated mitochondrial restoration and neuroprotection in primary neurons in an AD in vitro model. Further studies are on the way, focusing on the light-induced mitochondrial recovery in vivo and how to enhance its efficacy, with the goal of translating this approach to improve AD management.

## Figures and Tables

**Table 1:** Parameters of mitochondrial network and cellular physiology in cultured primary neurons after exposure to Aβ and/or PBM. The percentage changes for the PBM and Aβ groups were calculated taking values for the control group (untreated neurons) as 100%; the changes for the Aβ + PBM, Aβ + Mdivi-1, and Aβ + Mdivi-1 + PBM groups were calculated compared to the Aβ group. The “+” and “−” signs were placed before the values of statistically significant data to mark the direction of the changes.

Parameter		Experimental Groups					
		Control	PBM	Aβ	Aβ + PBM	Aβ + Mdivi-1	Aβ + Mdivi-1 + PBM
Mitochondrial network	Total count	788	953 (+21%)	2078 (+164%)	1462 (−79%)	1909	1952
	Total volume (μm^3^)	1672	2477 (+48%)	798 (−52%)	1483 (+41%)	982	758
	Average volume (μm^3^)	2.16	2.55 (+18%)	0.40 (−82%)	0.98 (+27%)	0.44	0.41
	Surface area (μm^2^)	11,944	16,439 (+38%)	8444 (−29%)	11,549 (+26%)	10,021	9480
	Average area (μm^2^)	15.18	17.30 (+14%)	4.19 (−72%)	7.94 (+25%)	4.23	4.19
	Total number of branches	5948	7853 (+32%)	2486 (−58%)	4162 (+28%)	2523	2593
	Average number of branches	7.95	8.28	1.28 (−84%)	3.14 (+23%)	1.37	1.35
	Total branch length (µm)	7524	11,340 (+51%)	2274 (−70%)	4162 (+25%)	2410	2296
	Average branch length/mito. (μm)	9.70	11.71 (+21%)	1.18 (−88%)	2.92 (+18%)	1.20	1.15
	Total number of branch junctions	3006	3786 (+26%)	1480 (−51%)	2295 (+27%)	1538	1517
	Average number of junctions	3.88	3.90	1.10 (−72%)	1.57 (+12%)	1.14	1.11
	Total number of branch endpoints	1903	1047 (−45%)	2747 (+44%)	2199 (−29%)	2814	2919
	Average number of endpoints	5.60	4.51 (−19%)	1.31 (−77%)	1.49	1.29	1.30
	Branch diameter (µm)	0.42	0.22 (−48%)	0.72 (+71%)	0.47 (−59%)	0.70	0.71
	Branch sphericity	0.04	0.04	0.14 (+258%)	0.09 (−128%)	0.13	0.14
Viability after 24 h		100%	100%	72% (−28%)	87% (+15%)	79% (+7%)	74%
Membrane depolarization (mV)	after 10 min	0	12	0	12	0	12
	after 24 h	0	0	21	14 (−33%)	17	19
CtROS	after 10 min	100%	118% (+18%)	100%	100%	100%	120% (+20%)
	after 24 h	100%	100%	144% (+44%)	126% (−18%)	136%	140%
MtROS	after 10 min	100%	115% (+15%)	100%	100%	100%	115% (+15%)
	after 24 h	100%	100%	136% (+36%)	124% (−12%)	129%	131%
ATP	after 10 min	100%	122% (+22%)	100%	122% (+22%)	100%	122% (+22%)
	after 24 h	100%	100%	62% (−38%)	81% (+19%)	68% (−32%)	70% (+8%)

## Data Availability

The original contributions presented in this study are included in the article/Supplementary Materials. Further inquiries can be directed to the corresponding authors.

## Supplementary Materials

The following supporting information can be downloaded at: https://www.mdpi.com/article/10.3390/ijms27177834/s1.

## Abbreviations

The following abbreviations are used in this manuscript:

AD	Alzheimer’s disease
Aβ	amyloid-β
ATP	adenosine triphosphate
CMP	cell membrane potential
CLSM	confocal laser scanning fluorescence microscopy
CCK	cell counting kit
CCO	cytochrome c oxidase
CtROS	cytosolic reactive oxygen species
DRP	dynamin-related protein
ER	endoplasmic reticulum
ETC	electron transport chain
MFN	Mitofusin
Mdivi	mitochondrial division inhibitor
MtMP	mitochondrial membrane potential
MtROS	mitochondrial reactive oxygen species
mPTP	permeability transition pore
NIR	near-infrared
OPA	optic atrophy protein
PBM	Photobiomodulation
p-tau	Protein
ROS	reactive oxygen species
3D	three-dimensional
