# Supplementary material for: Photobiomodulation with 808 nm Laser Light Repairs Mitochondrial Integrity and Normalizes ROS, ATP, and Membrane Depolarization in Aβ-Exposed Primary Neurons in Alzheimer’s Disease Model
Source: Int J Mol Sci. 2026 Sep 1;27(17):7834. doi: 10.3390/ijms27177834 (PMC13565913; doi:10.3390/ijms27177834)
Supplement: Supplementary file 1 [file ijms-27-07834-s001.zip › Supplementary Materials_IJMS-2.pdf]

Supplementary Materials

Photobiomodulation with 808 nm Laser Light Repairs Mitochondrial Integrity and Normalizes ROS, ATP, and Membrane Depolarization in Aβ-Exposed Primary Neurons in Alzheimer's Disease Model

Iuliia Golovynska [1,†], Binjun Li [1,†], Qinglin Chen [1], Sergii Golovynskyi [1], Xu Hao [1], Yurii V. Stepanov [2], Liudmyla I. Stepanova [3], Fangrui Lin [1,*], Junle Qu[1,*] and Tymish Y. Ohulchanskyy [1,‡]

[1] Shenzhen Key Laboratory of Photonics and Biophotonics, College of Physics and Optoelectronic Engineering, Shenzhen University, Shenzhen 518060, China; iuliia@szu.edu.cn (I.G.)

[2] R.E. Kavetsky Institute of Experimental Pathology, Oncology and Radiobiology, National Academy of Science of Ukraine, 03022 Kyiv, Ukraine

[3] Institute of Biology and Medicine, Taras Shevchenko National University of Kyiv, 01601 Kyiv, Ukraine

* Correspondence: frlin@szu.edu.cn (F.L.); jlqu@szu.edu.cn (J.Q.)

† These authors contributed equally to this work.

‡ Current address: Institute for Lasers, Photonics and Biophotonics and Department of Chemistry, University at Buffalo, Buffalo, NY 14260, USA.

Methods

Isolation of Primary Mouse Hippocampal Neurons

Primary mouse hippocampal neurons, isolated from postnatal C57BL/6J mouse pups (both male and female), were obtained from Guangzhou Huateng Biomedical Technology Co., Ltd., China. All animal procedures were performed in strict accordance with Experimental Animal Use Regulations of Guangdong Province Department of Science and Technology (permit No. SYXK-2024-0307) and the National Institutes of Health (NIH) Guidelines for the Care and Use of Laboratory Animals. The mice were maintained in collective cages under standard controlled conditions on a 12 h light/dark cycle and fed standard rodent chow and water ad libitum. Humane endpoints were established to minimize animal suffering throughout the experimental process.

Our selection of neonatal mouse hippocampal primary neurons over adult neurons, in vivo AD models, or commercial neuronal cell lines was driven by our core focus on quantitative characterization of PBM-mediated mitochondrial structural/functional restoration, a critical goal for this study's mechanistic analysis. Neonatal neurons yield high viability/purity and a well-differentiated mitochondrial network, enabling rigorous 3D confocal quantification of mitochondrial morphology and precise measurement of ROS, ATP, and membrane potential. On the contrary, adult neuron isolation causes irreversible mitochondrial damage (fragmentation and swelling) via enzymatic/mechanical dissociation, precluding reliable mitochondrial analysis. In vivo AD models introduce confounding systemic/nonneuronal variables (aging or neuroinflammation) that obscure direct Aβ/PBM effects on neuronal mitochondria. We avoided commercial neuronal cell lines because they lack the mature mitochondrial network, postmitotic phenotype, and physiological relevance of primary neurons, and harbor immortalization-induced genetic/metabolic alterations that distort AD-relevant mitochondrial dynamics, ROS regulation, and ATP production, making them an unreliable model for our precise mitochondrial analyses. Hence, primary neonatal neurons are a gold-standard, reproducible model that recapitulates Aβ-induced mitochondrial stress in a neuron-intrinsic system, ensuring our findings reflect native neuronal mitochondrial biology and establish a critical mechanistic foundation for translational research.

Animal specification and sacrificing method. Primary hippocampal neurons were isolated from postnatal day 0 (P0) to postnatal day 1 (P1) C57BL/6J mouse pups (both male and female). The average body weight of the pups at the time of tissue collection was 1.2–1.5 g. No adult mice were euthanized for this neuron isolation procedure. Euthanasia of mouse pups was performed using a two-step method combining anesthesia followed by a confirmatory physical method, in compliance with IACUC guidelines for neonatal rodents. First, pups were placed in a sealed glass chamber

pre-charged with 4% isoflurane (Sigma-Aldrich, USA) vaporized in medical-grade oxygen (flow rate: 1 L/min). Anesthesia was maintained for 3–5 min until the pups exhibited loss of righting reflex, cessation of pedal withdrawal response to toe pinch, and shallow irregular breathing, altogether confirming a deep plane of anesthesia. Following anesthesia induction, euthanasia was completed via cervical dislocation, performed using sterile forceps. Death was confirmed by the absence of spontaneous breathing, cardiac activity (assessed via palpation of the thoracic cavity), and corneal reflex for a minimum of 10 min prior to tissue dissection.

Protocol for isolation of mouse hippocampal neurons. The isolation and primary culture of hippocampal neurons were performed using P0–P1 C57BL/6J mouse pups (4–6 per culture, ~1–2 × 10^6 neurons/pup), yielding viable neurons for in vitro studies. Pup's brains were collected in cold Hanks' balanced salt solution (HBSS). Then a HBSS solution was replaced to 0.25% Trypsin-EDTA solution (300 µL/pup) and incubated during 12 min at 37 °C with periodically shaking. The digestion was stopped with 5× volume HBSS. Further steps were to resuspend tissue in a medium via gentle pipetting (10×), settle clumps, and collect supernatant (filtering through 40 µm strainer). Cells were centrifuged at 300g for 5 min, resuspended in a medium, and counted viable cells (trypan blue). The cells were plated at 1 × 10^6 cells per 35 mm dish and incubated 24 h for adhesion. Afterward, we replaced half a medium with a fresh medium post-adhesion and repeated this procedure every 3 days. Neurons maturated by 12 cultivation day and then were used for the experiments (Stefanie Kaech & Gary Banker. Culturing hippocampal neurons. *Nature Protocols*, volume 1, pages 2406–2415 (2006)).

Cell counting kit (CCK)-8 assay for cell viability detection

Cell viability was quantified via CCK-8 assay (Thermo Fisher Scientific, USA) following the manufacturer's instructions. Briefly, the cells were resuspended and seeded (1×10^4 cells/well) in 96-well plates with blank and replicate wells. After 24 h adhesion, the cells were incubated with Aβ for the next 24 h, and without Aβ for control evaluation. Further, 10 µL CCK-8 reagent was added to each well, incubated for 4 h. The assay is based on WST-8 reduction by viable cell dehydrogenases to form formazan, with absorbance at 450 nm (reference absorbance at 630 nm) correlating with viable cell number.

Western blot for mitochondrial dynamics proteins

At the 24 h experimental endpoint, whole-cell lysates were harvested from primary hippocampal neurons for immunoblot analysis of mitochondrial dynamics proteins. Samples were separated on 10% SDS-PAGE mini-gels, transferred to PVDF membranes, and probed with primary antibodies targeting OPA-1 (120 kDa), phosphorylated DRP1 (Ser616, ~80 kDa), and β-Actin (42 kDa) as a loading control. Due to lane number constraints, the samples were split across multiple independent gels; all target band intensities were normalized to the β-Actin signal from the same gel via ImageJ densitometry. Representative raw blots for all treatment groups are provided in Figure S2, and a consolidated composite blot summary is shown in Figure 4 in the main text.

Thresholding and contrast optimization in Mitochondrial Analyzer 3D

Mitochondrial segmentation was performed using the Mitochondrial Analyzer 3D plugin for ImageJ. Prior to thresholding, all z-stack images underwent consistent pre-processing: background subtraction (a rolling ball radius of 1.25 µm), sigma filter plus (a radius of 1.00 µm and a max slope of 1.40), enhanced local contrast (a scale factor of 2.600, applied between 0.50 and 0.80 of the full stack depth), and gamma correction (γ = 0.90). The weighted mean 3D local thresholding algorithm was employed. To minimize subjective threshold choice, the plugin 3D Threshold Optimize function was used to systematically screen block size and C-value parameters; upon visual comparison of segmented objects against raw fluorescence data, an optimized parameters block size of 1.05 µm and a C-value of 10 were adopted for all datasets. Post-segmentation processing included despeckling, outlier removal (a radius of 1.0 pixel) and 3D hole filling. Identical processing parameters were applied to all images across experimental groups.

Results

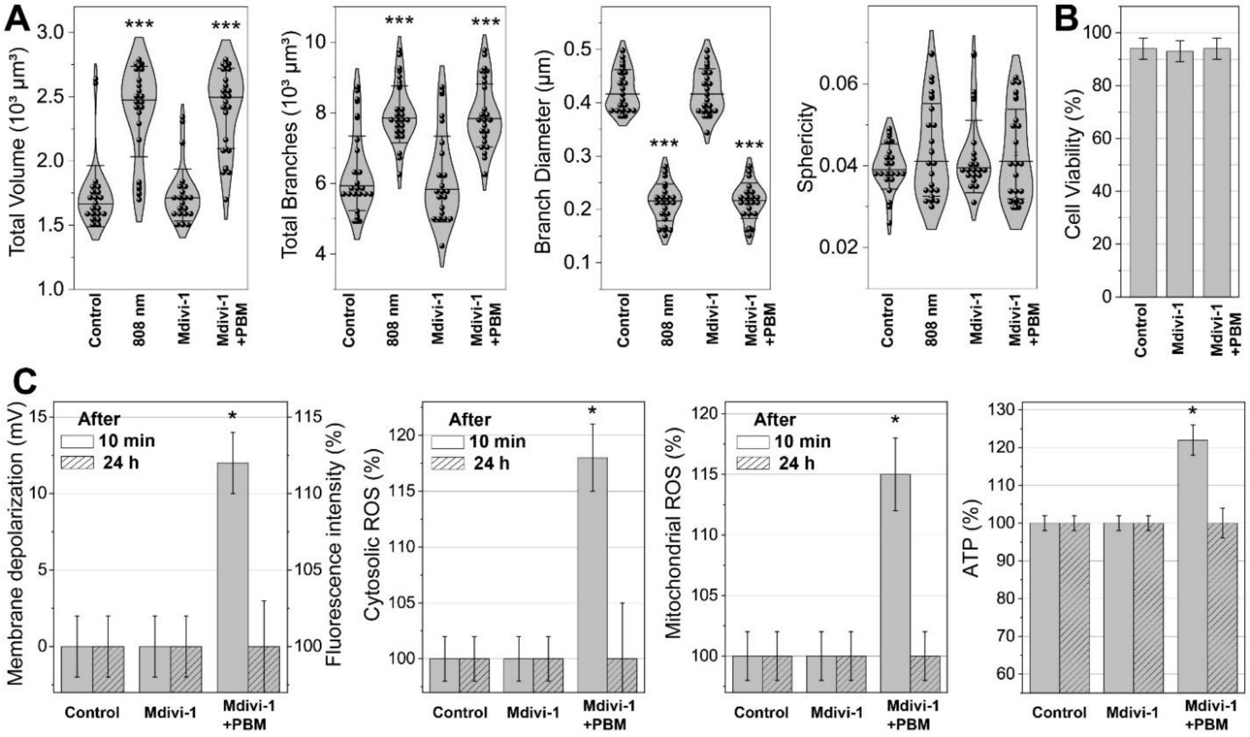

Figure S1. (A) The influence of Mdivi-1 and Mdivi-1+PBM (808 nm laser for 5 min, 100 mW/cm^2, 30 J/cm^2) on mitochondria network in healthy neurons, showing the 3D quantification of mitochondrial volume, branches, diameter and sphericity. **(B)** Cell viability of neurons after treatment with Mdivi-1 and PBM. **(C)** Impact of Mdivi-1 and PBM on neuronal CMP, ROS, and ATP production measured after 10 min and 24 h using epifluorescence microscopy of neurons stained with fluorescent probes. Data are presented as M ± SD, with $^{*}p < 0.05$ and $^{***}p < 0.001$ indicating statistically significant differences against the control.

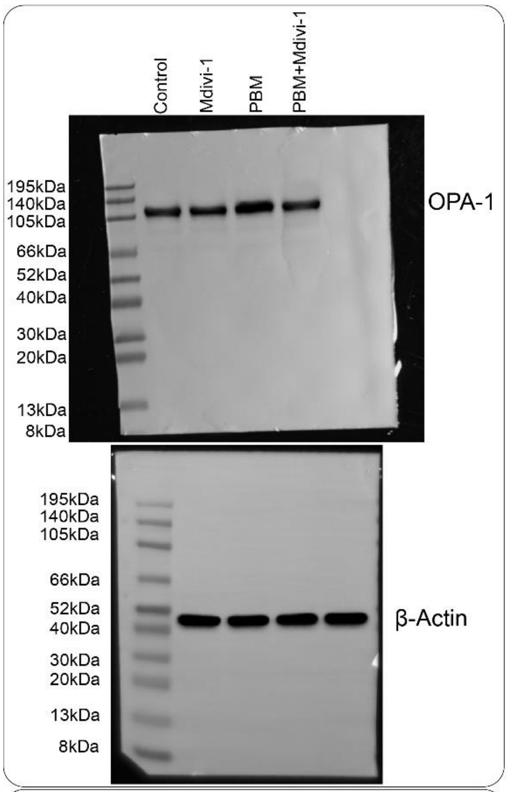
Control
Mdivi-1
PBM
PBM+Mdivi-1
195kDa
140kDa
105kDa
66kDa
52kDa
40kDa
30kDa
20kDa
13kDa
8kDa
OPA-1
β-Actin

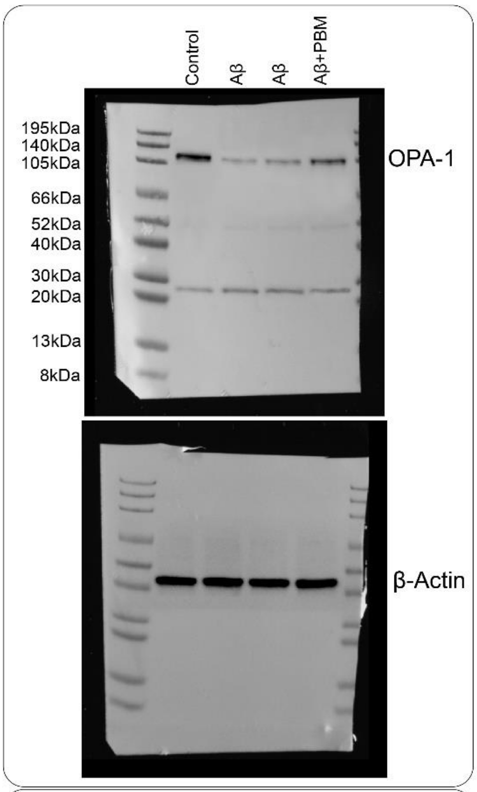
Control
Aβ
Aβ
Aβ+PBM
195kDa
140kDa
105kDa
66kDa
52kDa
40kDa
30kDa
20kDa
13kDa
8kDa
OPA-1
β-Actin

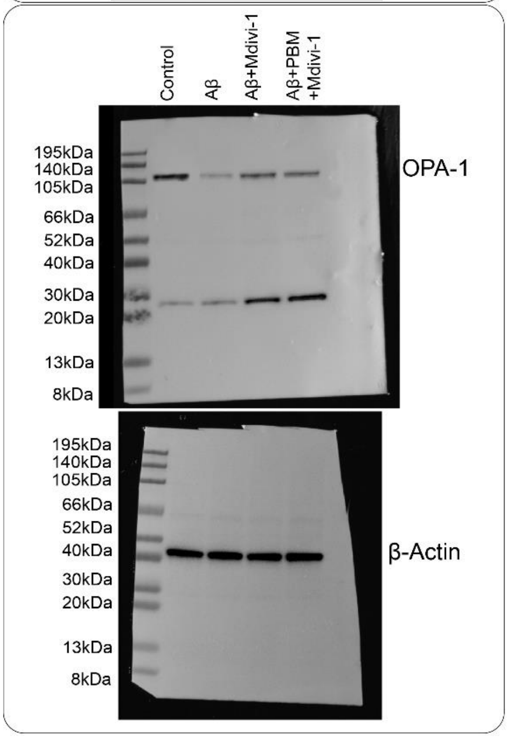
Control
Aβ
Aβ+Mdivi-1
Aβ+PBM
+Mdivi-1
195kDa
140kDa
105kDa
66kDa
52kDa
40kDa
30kDa
20kDa
13kDa
8kDa
OPA-1
β-Actin

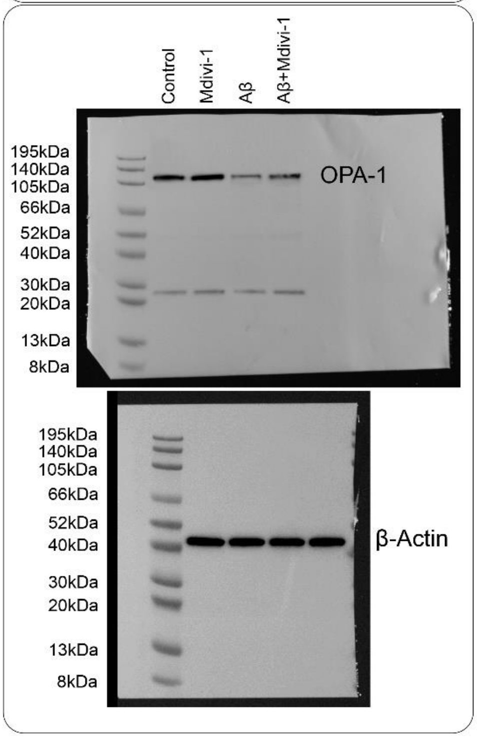
Control
Mdivi-1
Aβ
Aβ+Mdivi-1
195kDa
140kDa
105kDa
66kDa
52kDa
40kDa
30kDa
20kDa
13kDa
8kDa
OPA-1
β-Actin

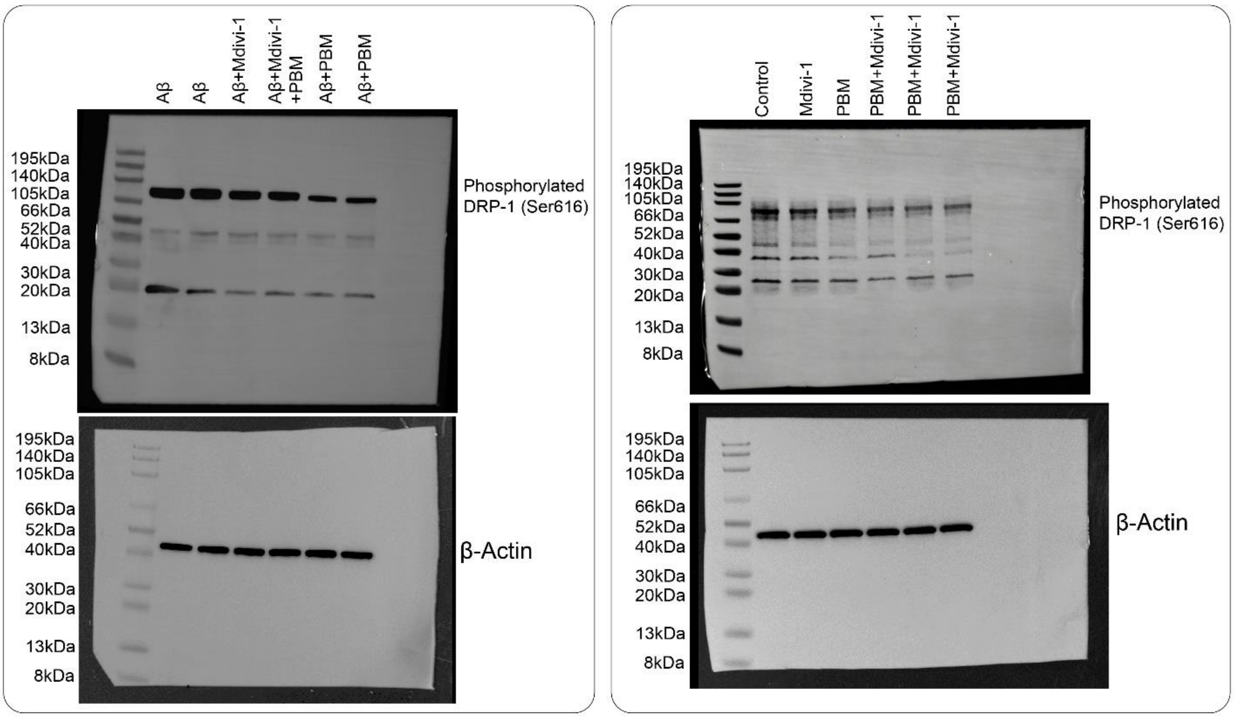

Figure S2. Western blot analysis of OPA-1 and phosphorylated DRP-1 (p-DRP-1, Ser616) protein expressions in primary mouse hippocampal neurons under different experimental treatments. Due to limited lane capacity of individual mini-gels, experimental samples were separated across multiple independent SDS-PAGE gels; all OPA-1 band intensities were normalized to the internal β-actin loading control measured within each respective gel for quantitative comparison. Representative raw blot images for each treatment group are shown, alongside a consolidated composite blot summarizing all experimental conditions.

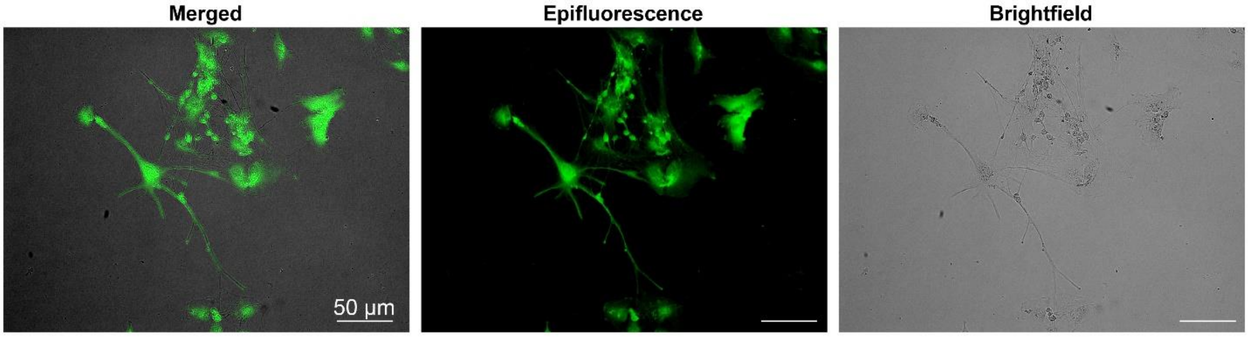

Figure S3. Aβ immunofluorescence/transmission microscopy images performed using labeling with oligomer-specific $A\beta_{1-42}$ antibody (green emission, 490/515 nm, Thermo Fisher Scientific, USA).
