# Supplementary material for: Photobiomodulation with 808 nm Laser Light Repairs Mitochondrial Integrity and Normalizes ROS, ATP, and Membrane Depolarization in Aβ-Exposed Primary Neurons in Alzheimer’s Disease Model
Source: Int J Mol Sci. 2026 Sep 1;27(17):7834. doi: 10.3390/ijms27177834 (PMC13565913; doi:10.3390/ijms27177834)
Supplement: Supplementary file 1 [file ijms-27-07834-s001.zip › Supplementary Materials_IJMS-2_WB analysis.pdf]

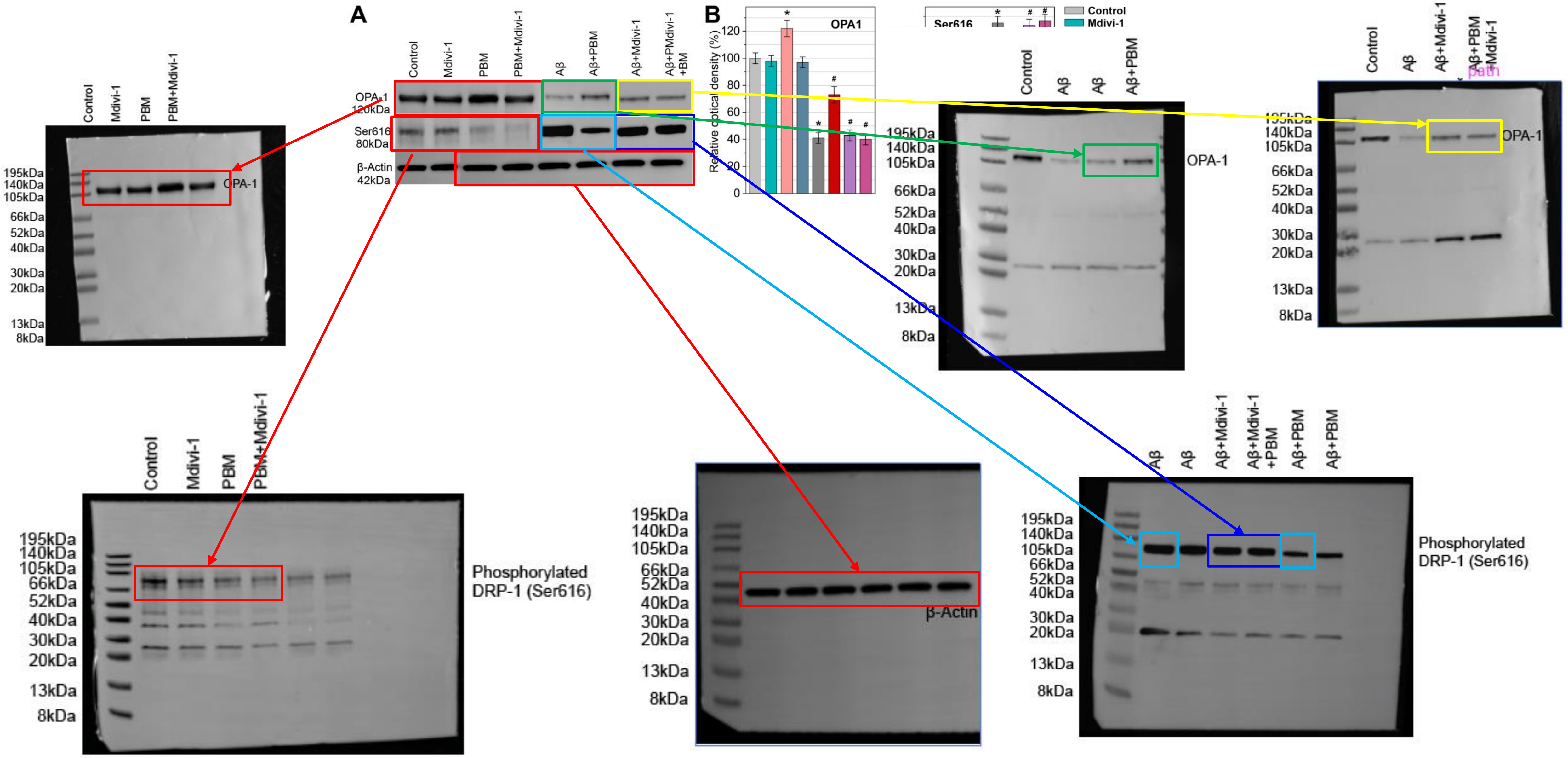

A
Control
Mdivi-1
PBM
PBM+Mdivi-1
Aβ
Aβ+PBM
Aβ+Mdivi-1
Aβ+PMdivi-1 +BM
OPA-1 120kDa
Ser616 80kDa
β-Actin 42kDa
B
OPA1
Relative optical density (%)
0
20
40
60
80
100
120
Ser616
Control
Mdivi-1
195kDa
140kDa
105kDa
66kDa
52kDa
40kDa
30kDa
20kDa
13kDa
8kDa
OPA-1
Phosphorylated DRP-1 (Ser616)
β-Actin
Aβ+PBM +Mdivi-1
Aβ+Mdivi-1 +PBM

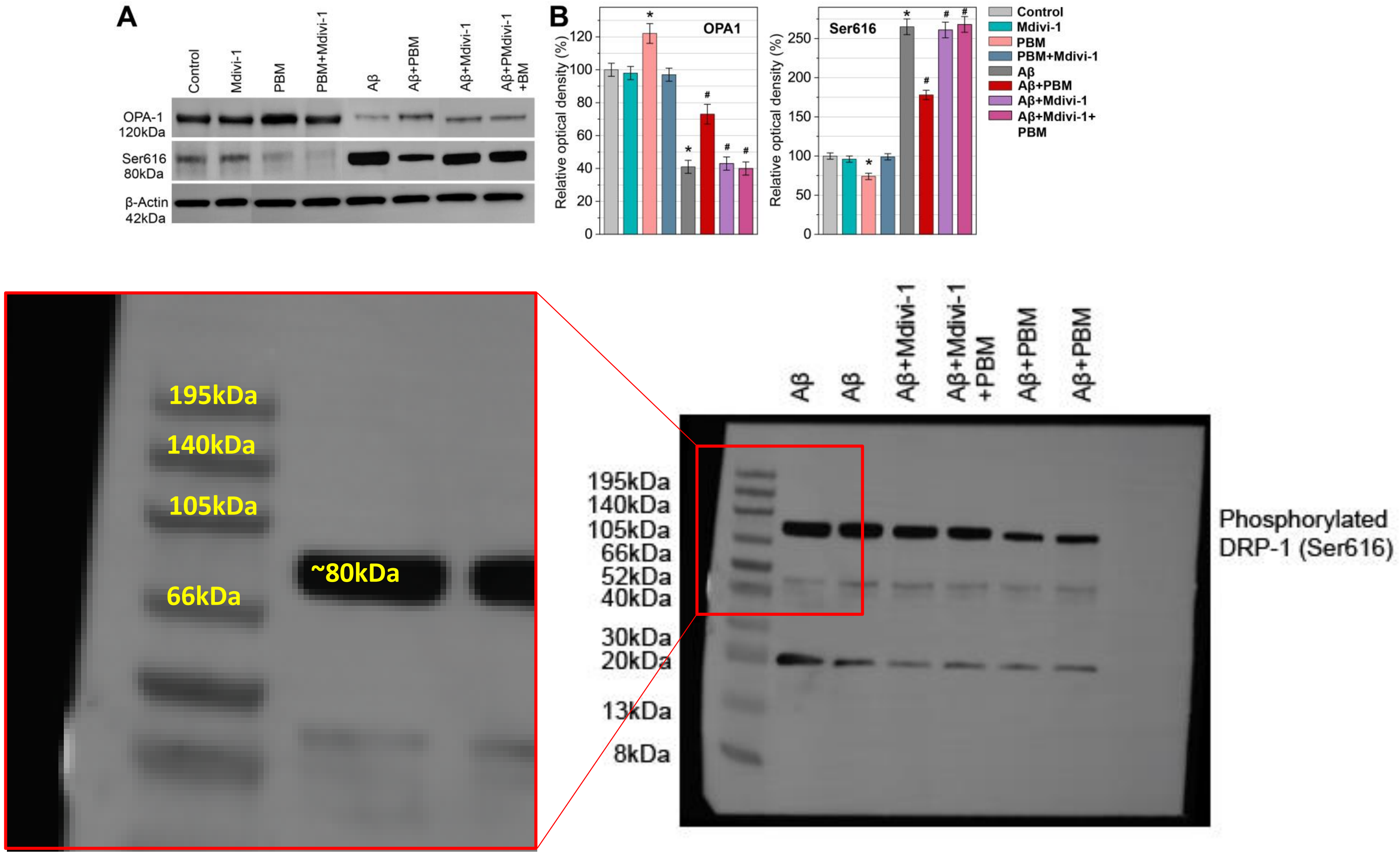
A
Control
Mdivi-1
PBM
PBM+Mdivi-1
Aβ
Aβ+PBM
Aβ+Mdivi-1
Aβ+PMdivi-1 +BM
OPA-1 120kDa
Ser616 80kDa
β-Actin 42kDa
B
OPA1
Relative optical density (%)
Ser616
Control
Mdivi-1
PBM
PBM+Mdivi-1
Aβ
Aβ+PBM
Aβ+Mdivi-1
Aβ+Mdivi-1+ PBM
195kDa
140kDa
105kDa
~80kDa
66kDa
Aβ
Aβ
Aβ+Mdivi-1
Aβ+Mdivi-1 +PBM
Aβ+PBM
Aβ+PBM
195kDa
140kDa
105kDa
66kDa
52kDa
40kDa
30kDa
20kDa
13kDa
8kDa
Phosphorylated DRP-1 (Ser616)

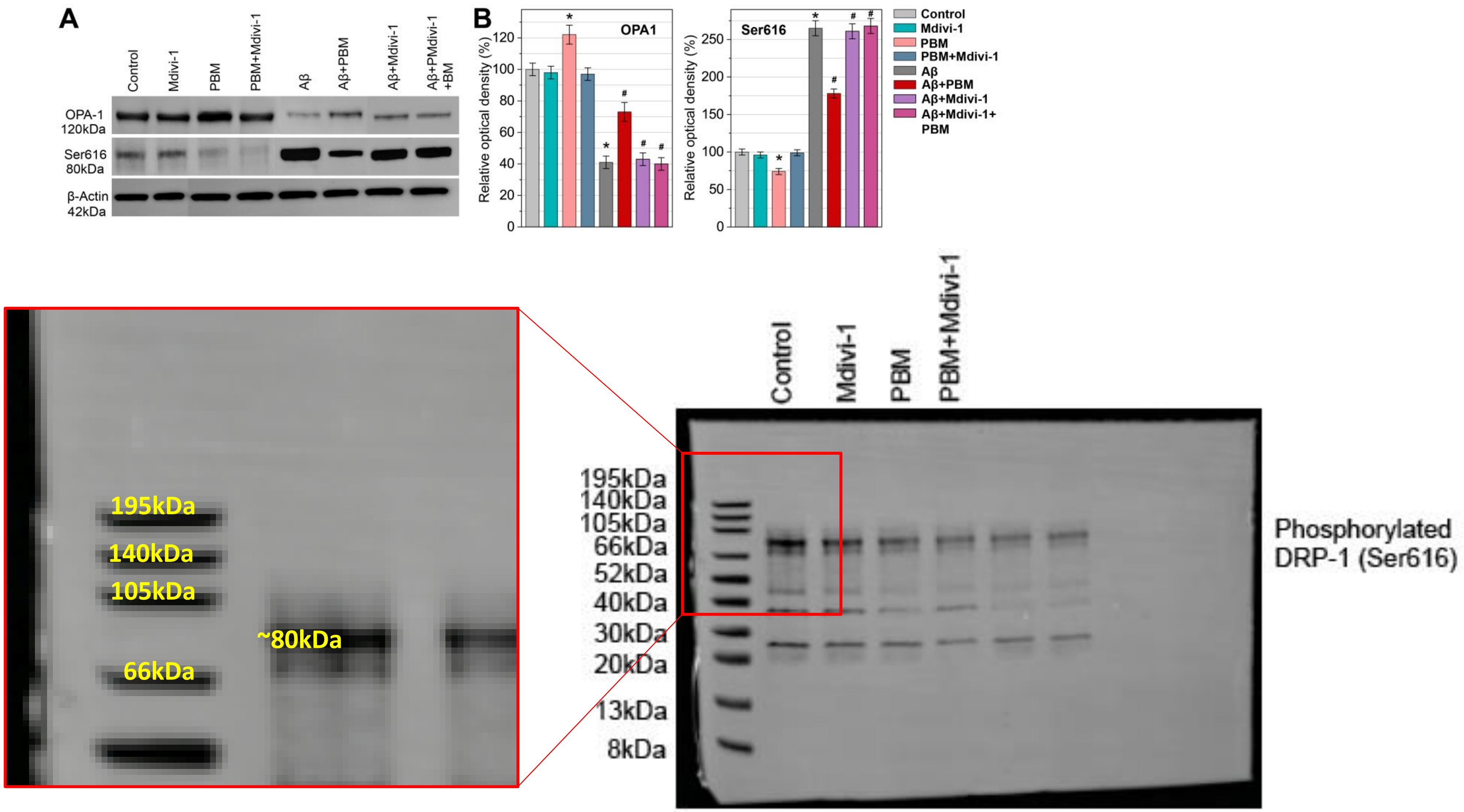

A
Control
Mdivi-1
PBM
PBM+Mdivi-1
Aβ
Aβ+PBM
Aβ+Mdivi-1
Aβ+PMdivi-1 +BM
OPA-1 120kDa
Ser616 80kDa
β-Actin 42kDa
B
OPA1
Relative optical density (%)
Ser616
Control
Mdivi-1
PBM
PBM+Mdivi-1
Aβ
Aβ+PBM
Aβ+Mdivi-1
Aβ+Mdivi-1+ PBM
195kDa
140kDa
105kDa
~80kDa
66kDa
Control
Mdivi-1
PBM
PBM+Mdivi-1
195kDa
140kDa
105kDa
66kDa
52kDa
40kDa
30kDa
20kDa
13kDa
8kDa
Phosphorylated DRP-1 (Ser616)
